# Pectinase Immobilization on Porous Polyamide Microparticles: Characterization, Operational Stability and Application in Wine Clarification

**DOI:** 10.3390/molecules31162930

**Published:** 2026-08-21

**Authors:** Sandra C. Oliveira, Nadya V. Dencheva, Zlatan Z. Denchev

**Affiliations:** IPC—Institute for Polymers and Composites, Campus of Azurém, University of Minho, 4800-058 Guimarães, Portugal; b8223@dep.uminho.pt (S.C.O.); nadiad@dep.uminho.pt (N.V.D.)

**Keywords:** wine clarification, lyophilized pectinase, noncovalent enzyme immobilization, PA6 microparticulate carriers, biocatalyst reusability, Michaelis–Menten kinetics

## Abstract

Lyophilized pectinase from *Aspergillus niger* (PeL) was immobilized onto polyamide 6 (PA6) microparticles (MPs) through an adsorption-based procedure within the pH range of 5–8, yielding four PeL@PA6 complexes. In contrast to commercial enological preparations that are complex enzymatic cocktails with unspecified exact compositions, the use of PeL with known specific activity enabled a more reliable evaluation and improvement of the immobilization process and of the structure–activity relationships of the resulting biocatalysts. Thermogravimetric analysis demonstrated better thermal stability of the PeL@PA6 complexes compared to neat PA6 MPs. UV-CD studies revealed that the secondary structure and conformational stability of PeL before and after immobilization were strongly pH-dependent, with maximum stability observed at pH 6–7. All four PeL@PA6 complexes retained significant catalytic activity and showed good tolerance to ethanol-rich media relevant to enological applications. Kinetic analysis indicated increased apparent *K_m_* values after immobilization, suggesting diffusional limitations associated with the porous PA6 support. Clarification experiments of industrial white and rosé wine musts confirmed the practical applicability of the immobilized system. All PeL@PA6 complexes preserved the color and phenolic integrity of the musts, displayed good operational stability during reuse, and exhibited higher long-term storage stability than the free enzyme. These results demonstrate that PA6 MPs are promising supports for pectinase immobilization in wine clarification and related biotechnological applications.

## 1. Introduction

Enzymes play an important role at various stages of wine production, whereby many of them originate naturally from the grapes themselves [1]. However, endogenous grape enzymes are often present in insufficient quantities and may not exhibit optimal activity under industrial winemaking conditions. Therefore, the use of commercial enzyme preparations in various vinification stages, e.g., maceration, clarification, aroma release, or filtration, has become a well-established practice in modern winemaking, particularly in large-scale production [2].

Clarification is a crucial step in vinification, aimed at reducing turbidity in grape must and wine. This turbidity is mainly caused by high-molecular-weight pectic polysaccharides originating from grape cell walls and released into the must during crushing and pressing. These substances form colloidal suspensions that hinder sedimentation and filtration, thereby affecting processing efficiency and the visual quality of the final wine. For this reason, the addition of exogenous pectinolytic enzymes is often necessary to improve clarification, filtration, and must yield [3,4,5]. 

Clarification is particularly important in the production of white and rosé wines. In white wine production, the must is separated from the skins immediately after crushing, which limits the extraction of phenolic compounds and structural polysaccharides. In contrast, rosé wines are produced with a short maceration period, which increases the extraction of cell wall-derived polysaccharides and colloidal material, resulting in higher turbidity and more complex clarification and stabilization requirements compared to white wine must [6].

Commercial pectinolytic enzyme preparations used for large-scale must clarification include products such as Rapidase (DSM, Delft, The Netherlands), Pectinex (Novonesis, Bagsværd, Denmark), Lallzyme (Lallemand, Montreal, QC, Canada), Scottzyme (Scott Laboratories, Petaluma, CA, USA), or Viazym (Martin Vialatte, Magenta, France). These preparations are multienzyme liquid complexes produced from *Aspergillus niger* fermentation broths and contain a poorly defined mixture of polygalacturonase (E.C. 3.2.1.15), pectin lyase (E.C. 4.2.2.10), pectin methylesterase (E.C. 3.1.1.11), and auxiliary activities from cellulases and hemicellulases. The synergistic action of this whole enzymatic system enables efficient industrial degradation of complex pectic polysaccharides [7]. Although these commercial multienzyme preparations have been successfully immobilized using a wide variety of supports and immobilization techniques, it should be noted that their exact composition and the relative activity of the individual enzymes in these commercial preparations are not disclosed by the manufacturers. Consequently, accurate quantification of the immobilized protein, discrimination between the contributions of the individual enzymatic activities, and rigorous kinetic analysis of the immobilized biocatalyst become more challenging. In contrast, lyophilized pectinase preparations with known specific enzymatic activity are particularly suitable for fundamental immobilization studies, although their high cost makes large-scale industrial application economically impractical.

In industry, pectinase biocatalysts are most frequently applied in their free form. However, free enzymes often lack sufficient operational stability and are difficult to recover and reuse [8]. Moreover, the use of free enzymes may complicate the implementation of continuous and automated processes [9]. To overcome these limitations, immobilization of enological enzymes has been suggested. The comprehensive review by Ottone et al. [4], together with other more general studies [10,11], reports numerous examples of immobilized enzymes used at different stages of wine production. Enzyme immobilization can be performed on various supports, including natural or synthetic polymers and inorganic materials, using physical methods (e.g., adsorption, entrapment) or chemical methods (e.g., covalent binding to insoluble carriers, formation of cross-linked enzyme aggregates). Generally, these approaches lead to improved enzyme stability, easier separation of the biocatalyst from the treated medium, and the possibility of repeated use of the same enzymatic preparation.

Among the materials used as immobilization supports for pectinases, polyamides have attracted particular attention because they are considered very close synthetic analogs to protein matrices and can be synthesized in various forms, including fibers, films, powders, and microparticles. Accordingly, PA6 and PA66 have frequently been used as supports for pectinase immobilization, predominantly through covalent attachment after activation with agents such as glutaraldehyde or dimethyl sulfate [12,13,14,15,16,17]. However, these activation agents are toxic, which considerably limits the applicability of the resulting biocatalysts in the food industry.

These limitations can be overcome by the noncovalent, adsorption-based immobilization of pectinases onto polyamide microparticles with scaffold-like surface morphology. Thus, polyamide microparticles (PA MPs) with various particle shapes, sizes, and surface topologies based on PA4, PA6, PA12 homopolymers, and the copolymeric PA612, can be synthesized by activated anionic ring-opening polymerization (AAROP) of the corresponding lactams. In our previous studies, these microparticles were successfully used to immobilize Viazym^®^, a commercial multienzyme pectinase preparation, yielding reusable biocatalysts for rosé must clarification [18,19]. Among the investigated supports, PA6 exhibited the best overall performance.

Despite these advances, no studies have reported the adsorption immobilization of a lyophilized pectinase preparation with well-defined polygalacturonase activity (PeL) onto porous PA MPs, nor has the performance of such immobilized biocatalysts been evaluated in the clarification of real industrial musts. Furthermore, the use of a well-defined PeL preparation facilitates accurate quantification of the immobilized protein and enables more reliable interpretation of enzyme activity and kinetic parameters than commercial multienzyme formulations of undisclosed composition.

Based on these considerations, we hypothesized that adsorption immobilization of a commercial lyophilized pectinase onto porous PA6 microparticles would produce reusable biocatalysts with preserved catalytic activity and operational stability, suitable for efficient clarification of industrial grape musts, while simultaneously enabling a more rigorous characterization of the immobilization process and enzyme performance.

Thus, the objectives of the present work were (i) to improve the adsorption immobilization of lyophilized pectinase onto porous PA6 MPs by varying pH and enzyme concentration; (ii) to characterize the resulting biocatalysts by spectroscopic, thermal, activity, and kinetic analyses; and (iii) to evaluate their practical applicability in the clarification of real industrial white and rosé grape musts.

## 2. Results

### 2.1. Noncovalent Immobilization of PeL on PA6 MP

**Figure 1:**
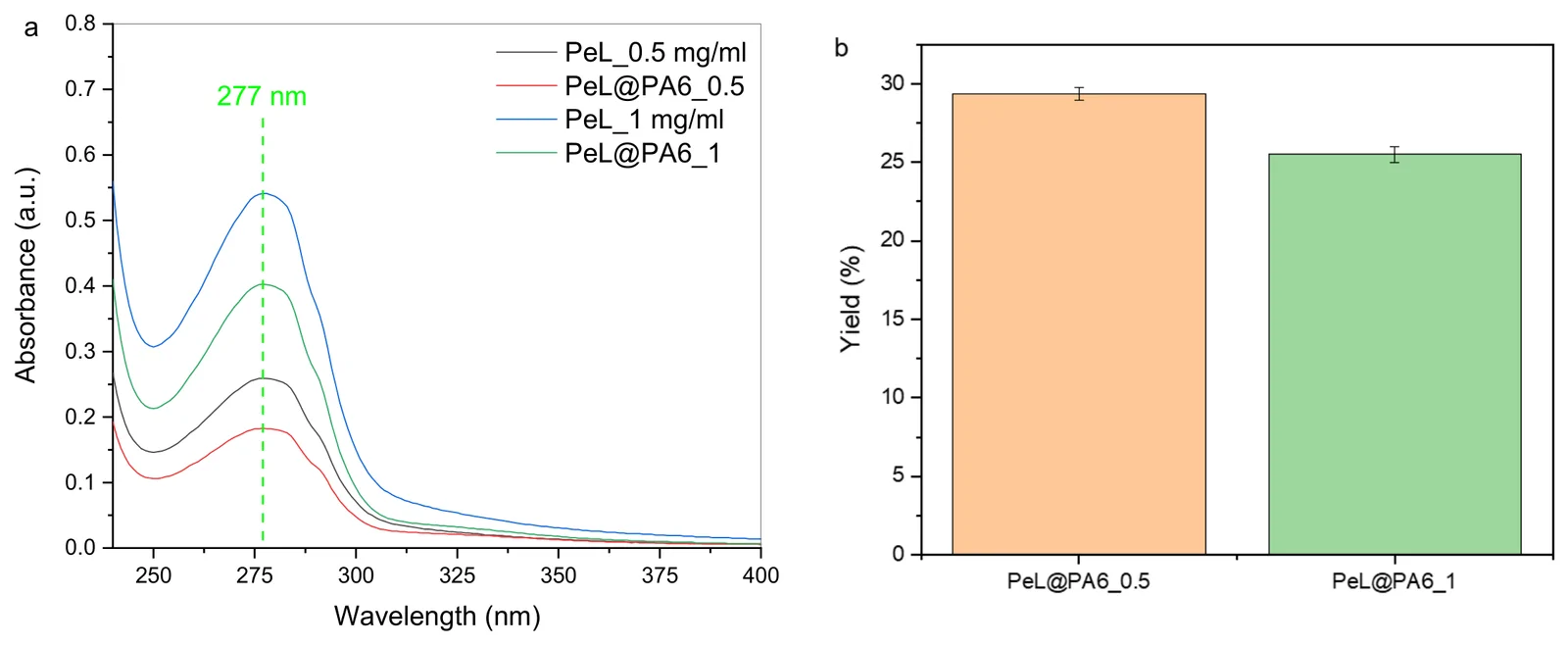
Quantification of the PeL immobilization: (**a**) UV/VIS spectra of the PeL solutions before and after immobilization, and (**b**) immobilization efficiency of PeL@PA6, where 0.5 corresponds to a pectinase concentration of 0.5 mg/mL, and 1 corresponds to a pectinase concentration of 1 mg mL^−1^. Standard deviations were based on two independent trials.

A series of experiments was conducted to identify the most suitable enzyme concentration and pH. Initially, the same amounts of PA6 MPs were incubated at pH = 7 (phosphate buffer) with two PeL concentrations (0.5 and 1.0 mg/mL). For clarity, these initial concentrations correspond to 25 and 50 mg enzyme g^−1^ PA6 MP support, respectively. After 24 h, the amount of non-immobilized enzyme remaining in the supernatant was quantified by UV–VIS spectroscopy at 277 nm, corresponding to the absorption of aromatic amino acid residues, mainly tryptophan and phenylalanine, present in the PeL molecules. The spectra of the pectinase solutions before and after immobilization and the corresponding immobilization yields (IY) calculated according to Equation (1) in Section 3 are presented in Figure 1a,b. 

The IY values obtained with the two enzyme concentrations were similar, in the range of 26–29%, with a slight decrease observed at the higher PeL concentration. These results suggest that, under the selected immobilization conditions, the adsorption capacity of the PA6 MPs approaches saturation already at 0.5 mg/mL; therefore, duplicating the initial enzyme concentration does not affect protein loading significantly. On the other hand, reducing the PeL concentration below 0.5 mg mL^−1^ resulted in immobilization yields of only 10–13% under the present experimental conditions, indicating that lower enzyme concentrations are not advantageous for efficient immobilization. Consequently, subsequent optimization experiments were carried out using 0.5 mg/mL PeL, keeping the same PA6 MP amount. 

**Figure 2:**
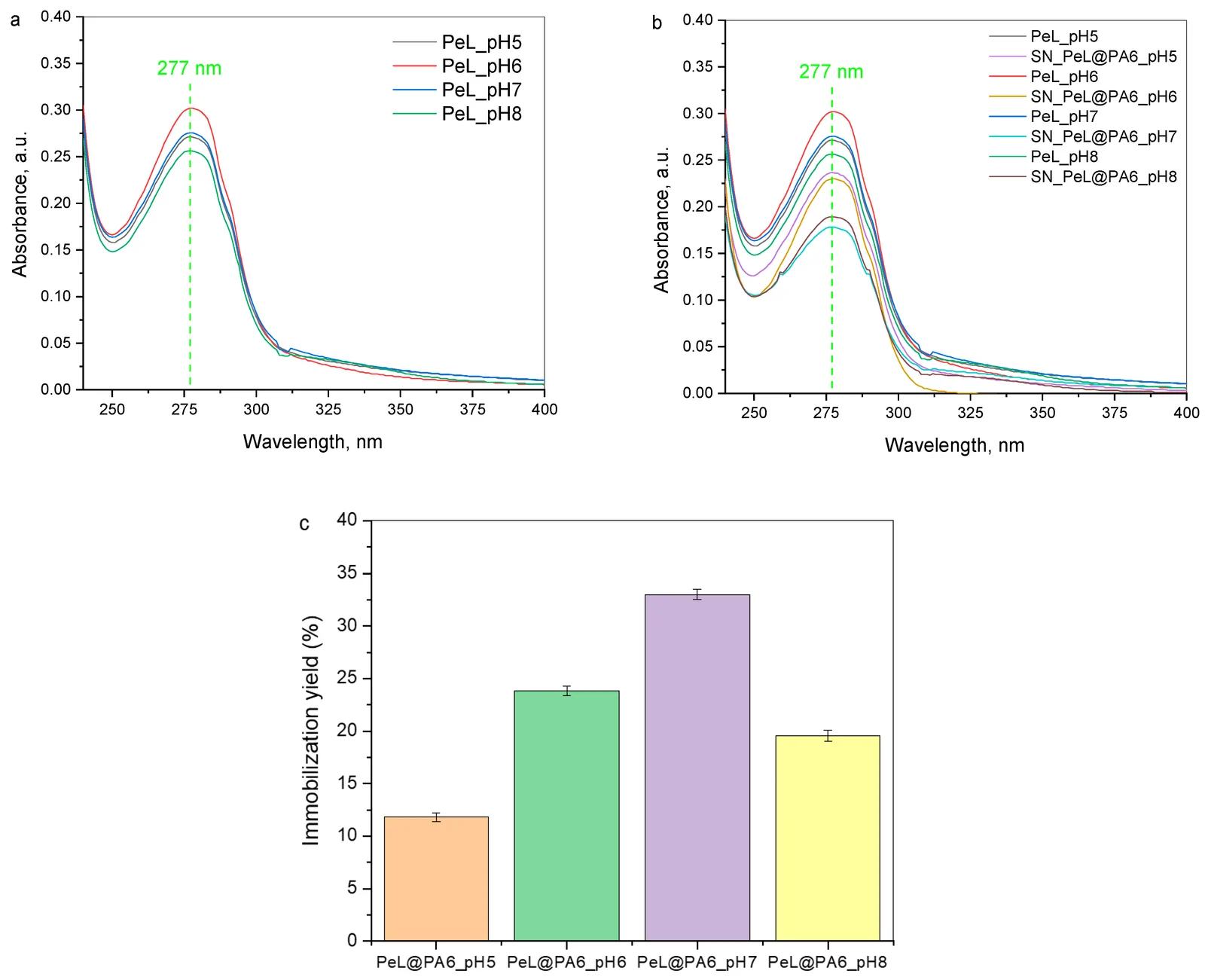
Quantification of the PeL immobilization: (**a**) UV/VIS spectra of the initial pectinase solutions; (**b**) UV/VIS spectra of the PeL solutions before and after immobilization; and (**c**) immobilization yield of PeL@PA6 at different pH values. Standard deviations were based on two independent trials.

The influence of pH on immobilization yield was investigated in the pH range 5–8 using the corresponding citrate or PB solutions. The UV–VIS spectra of the pectinase solutions before immobilization (e.g., PeL_pH5) and after immobilization in the respective supernatants (e.g., SN_PeL@PA6_pH5), as well as the immobilization yields, are shown in Figure 2.

The absorbance spectra of the initial PeL solutions exhibit clear differences in intensity as a function of pH (Figure 2a), indicating that the solubility, aggregation state, and/or conformational organization of the lyophilized PeL are pH-dependent. This behavior is expected considering the composition of this enzyme, comprising predominantly polygalacturonases (PG). The isoelectric point (pI) of PG is typically in the range of pH 3.5–5.0, and the pH optima for all five endo-polygalacturonases produced by *Aspergillus niger* were found in the range of 4.3–4.9 [20]. 

The maximum absorbance observed at pH 6 (Figure 2a, red curve) suggests improved PeL dispersion and greater structural stability, whereas the lower absorbance at pH 8 (blue curve) is consistent with conformational changes and/or partial protein aggregation.

The immobilization yields of PeL strongly depended on pH, reaching a minimum at pH 5 and a maximum at pH 7 (Figure 2c). This trend does not directly follow the absorbance profiles of the enzyme solutions, since the absorbance values at pH 5 and 7 are similar (black and green curves, Figure 2a). The observed differences can be rationalized considering the surface charge characteristics and colloidal stability of both the enzyme and the PA6 MP support. Due to their zeta potential, PA6 MPs are negatively charged at both pH 5 and 7, with reported values of −20.9 and −28.6 mV, respectively [21]. Above its pI, the PeL enzyme will also carry a net negative charge, becoming increasingly negative at higher pH values. Near pH 4–5, however, the net charge of the enzyme approaches zero.

The superior immobilization yield observed at pH 7 can therefore be attributed to the enhanced colloidal stability of both the PeL enzyme and the PA6 MP support under these conditions. The stronger negative zeta potential values at pH 7 likely reduce aggregation of both suspended enzyme molecules and polymer microparticles, thereby maximizing the accessible interfacial area and favoring effective enzyme–support contact. In contrast, at pH 5, the proximity of the enzyme pH to its pI, combined with the lower magnitude of the PA6 zeta potential, favors aggregation phenomena and reduces effective adsorption, despite the lower electrostatic repulsion between the enzyme and the support.

These results demonstrate that adsorption immobilization of pectinase onto PA6 MPs is not hindered by the moderate electrostatic repulsion between the negatively charged enzyme molecules and the negatively charged MP surface in the pH range 6–8. Hydrogen bonding between the enzyme and the PA6 surface can still occur because such interactions depend primarily on local functional groups rather than on the net surface charge. 

PA6 contains a high density of surface amide groups (–CONH–), which can act simultaneously as hydrogen-bond donors and acceptors. Likewise, pectinase molecules expose multiple hydrogen-bonding sites on their surface, including peptide backbone groups and polar amino acid side chains. In addition, hydrophobic interactions between nonpolar protein domains and the methylene-rich segments of PA6 may further contribute to adsorption. As a result, effective adsorption can occur even in the absence of favorable electrostatic attraction. Similar behavior was reported by Meissner et al. [22], who observed protein adsorption onto negatively charged silica nanoparticles both below and above the protein pI. On the other hand, our previous synchrotron X-ray studies on noncovalently immobilized laccase on PA6 MP supports [21] clearly proved intensive H-bond formation at the enzyme/MP interface.

The sodium phosphate buffers used in all immobilization experiments (excluding pH5, where a citrate buffer was applied) could attenuate long-range electrostatic interactions through ionic screening, thereby increasing the relative contribution of short-range interfacial interactions, including hydrogen bonding. The highly porous structure of the PA6 MPs, described in detail in previous SEM studies [18,19,21], further promotes enzyme adsorption by increasing enzyme–support contact and prolonging the residence time within the pores, thus enabling efficient interfacial interactions. Therefore, within the investigated pH range, the immobilization of PeL onto PA6 MPs can be described predominantly as a hydrogen-bond-driven adsorption process, while pH mainly modifies the balance between electrostatic repulsion, colloidal stability, and the geometry of the interfacial interactions.

#### 2.1.1. Circular Dichroism Analyses Before and After PeL Immobilization

Circular dichroism (CD) spectroscopy in the UV region of 190–250 nm probes electronic transitions of the peptide backbone that are highly sensitive to protein secondary structure. Distinct structural motifs, such as α-helices, β-sheets, β-turns, and unordered conformations, produce characteristic spectral signatures, enabling structural discrimination [23]. 

In the present study, comparative far-UV CD analysis was performed on the PeL solutions prior to immobilization and on the supernatants remaining after removal of the PeL@PA6 complexes, using pH as the variable parameter. Comparison of these spectra enabled evaluation of pH-induced conformational changes and assessment of possible structural alterations associated with enzyme–support interactions [24]. Quantitative secondary structure contents were determined by deconvolution of the experimental spectra using the CONTIN-LL algorithm and Reference Set 4 available in the DichroWeb online server as previously indicated [25]. 

The percentages of the different secondary structural elements are summarized in Table 1. The respective CD UV-VIS spectra and their fitting are presented in Figures S1–S3 of the Supplementary Materials.

As shown in Table 1, no usable CD data were obtained for the solutions at pH 5. Prior to CD measurements, all protein solutions were diluted to maintain the absorbance and detector high-tension (HT) voltage within the recommended operating range of the spectropolarimeter. The dilution factor was adjusted individually for each sample according to its protein concentration and optical properties. The samples prepared in citrate buffer at pH 5 required substantially greater dilution than those at pH 6–8 to achieve acceptable HT values. Consequently, the final protein concentrations (approximately 0.020 and 0.017 mg mL^−1^ for the initial PeL solution and the corresponding supernatant, respectively) produced an insufficient signal-to-noise ratio for reliable deconvolution of the CD spectra. Therefore, quantitative estimation of the secondary-structure contents was not possible for the pH 5 samples.

Overall, the far-UV CD data demonstrate systematic changes in the secondary structure of pectinase when comparing the initial enzyme solutions at different pH values with the corresponding supernatants obtained after immobilization. These results indicate that interaction between the enzyme and the PA6 MP support during immobilization alters the secondary structure of the non-immobilized enzyme fraction remaining in solution.

At all investigated pH values, the α-helix content increased after immobilization. For example, at pH 6 the α-helix fraction approximately doubled, increasing from 12.8 to 25.8%, whereas at pH 8 an even larger relative increase (≈477%) was observed. Simultaneously, the β-sheet content decreased markedly, from 37.7 to 11.4% at pH 6 (≈70% decrease) and by approximately 63% at pH 8. Smaller increases were detected for turns and unordered structures, typically by 4–6% and 3–10%, respectively.

The most pronounced trend revealed by the CD data is the apparent conversion of β-sheet structures into α-helical conformations after prolonged contact with the PA6 MPs. Such changes are consistent with conformational adaptation upon interaction with the PA6 surface. α-Helices are generally more compact and thermodynamically stable than extended β-sheet structures, and an increase in α-helix content may indicate adoption of a more stable conformation caused by the support surface. In general, α-helices tolerate structural perturbations, including environmental changes, better than β-strands, suggesting greater conformational robustness [26]. Accordingly, the decrease in β-sheet content accompanied by an increase in α-helix content may reflect partial reorganization of β-structured segments into more compact conformations promoted by interfacial hydrogen bonding and local packing constraints at the PA6 MP surface. Similar trends were reported by Burgos et al. [27], who observed changes in secondary-structure distribution after enzyme encapsulation and attributed them to confinement effects.

The observed β-sheet/α-helix redistribution reflects structural differences within the enzyme population remaining in solution and may result from both selective adsorption and solution-phase conformational re-equilibration. Most probably, the PA6 MP carrier preferentially immobilizes PeL molecules with higher β-sheet exposure, i.e., possibly more flexible, partially unfolded, aggregated, or surface-interacting conformers. Their removal from solution leaves a supernatant enriched in more α-helical and turn-containing conformations. The stronger effect at pH 8 suggests that the adsorption selectivity increases with pH, likely due to changes in PeL charge distribution, conformational flexibility, and/or hydrophobic interactions with the PA6 surface.

Overall, the CD results indicate that the PeL enzyme undergoes measurable secondary-structure changes during immobilization, depending on both pH and interaction with the PA6 MPs. Such structural alterations should influence the catalytic activity and stability of the immobilized PeL@PA6 conjugates, as discussed in the following sections. It should also be noted that relatively few far-UV CD studies addressing conformational changes in major pectinase classes are available in the literature, including reports on pectin methyl esterase [28], pectate lyases C and E [29], and exo-polygalacturonase [30].

#### 2.1.2. Thermal Analyses by TGA

**Figure 3:**
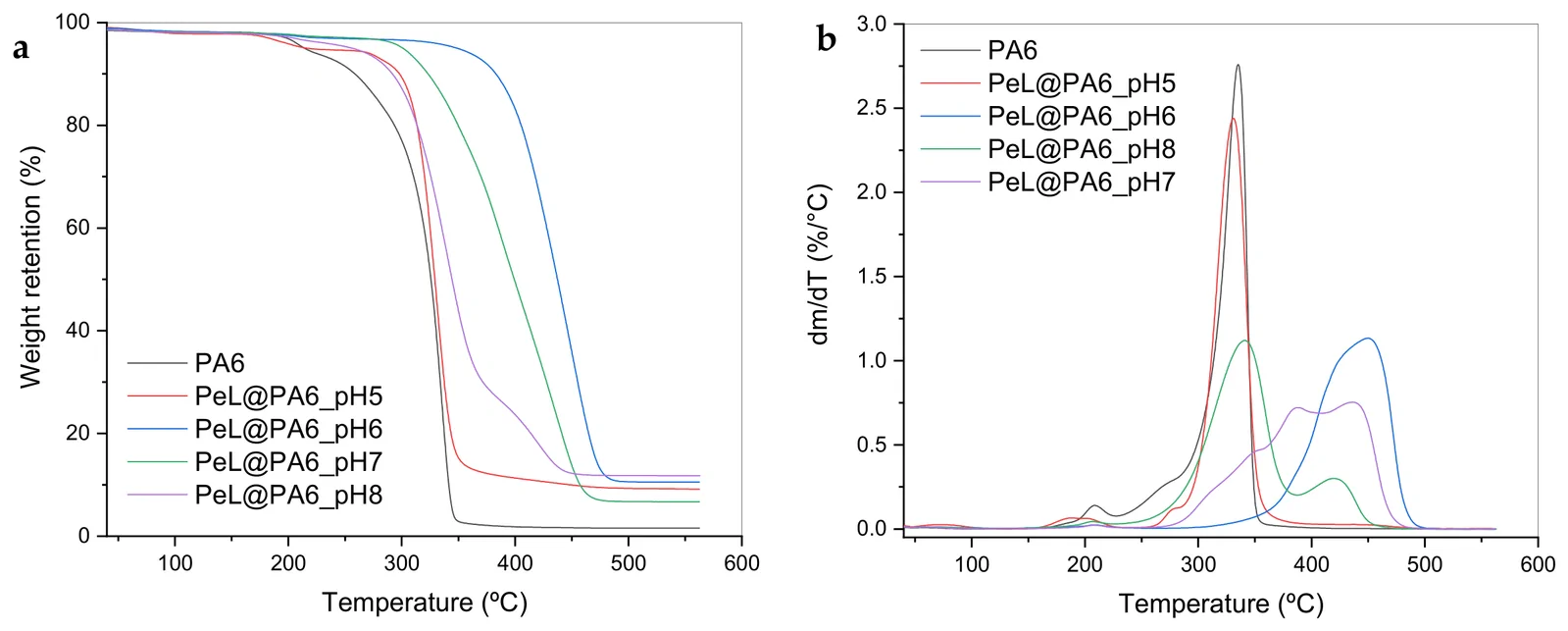
TGA curves of MP: (**a**) Integral weight loss curves and (**b**) first derivative curves.

To evaluate whether the thermal stability of the microparticles was affected by enzyme immobilization under different pH conditions, thermogravimetric analysis (TGA) was performed on PA6 MPs before and after PeL immobilization. The thermogravimetric curves, presented in both integral and differential forms, are shown in Figure 3, while the corresponding numerical parameters are summarized in Table 2.

The thermal behavior of the samples, illustrated in Figure 3a,b, reveals clear differences between neat PA6 MPs and the immobilized systems (PeL@PA6), confirming that the presence of pectinase modifies the thermal stability of the PA6 support. Neat PA6 MPs exhibit the typical thermal degradation profile of this thermoplastic polymer, characterized by a single major degradation event with an onset temperature *T_in_* of 267 °C, a maximum degradation temperature *T_max_* of 335.38 °C, a maximum degradation rate $V_{max}^{d}$ of 2.76%/min, and a low residual mass of 1.546% at 600 °C (Table 2). Moreover, the immobilized systems exhibit higher initial thermal resistance than neat PA6 MPs, as evidenced by their higher $T_{in}$ values. Among them, PeL@PA6_pH6 displays the highest thermal stability, with a $T_{in}$ value of 396.69 °C. These results suggest that the immobilized enzyme contributes to thermal stabilization of the microparticles and that the immobilization pH influences the nature of the enzyme–support interactions.

The differential TGA curves of the immobilized samples (Figure 3b) show multiple degradation stages, best expressed in samples PeL@PA6_pH6-8, which can be attributed to overlapping thermal decomposition processes of the enzyme and the polymer matrix. Furthermore, all immobilized systems exhibit lower $V_{max}^{d}$ values than neat PA6 MPs, indicating a more gradual degradation process. An increase in the residual mass at 600 °C due to char formation is also observed for all enzyme-containing samples (6.7–11.8%), compared with neat PA6 MPs (1.546%). This increase reflects the presence of immobilized pectinase without correlating quantitatively with enzyme loading.

Most likely, the degradation behavior of the most PeL@PA6 complexes originates from both enzyme carbonization and stabilization of the PA6 matrix. Like most proteins, pectinase contains nitrogen- and sulfur-containing amino acids, whose decomposition products are known to promote flame-retardant behavior in polymers, including polyamides [31]. Such compounds generally act by promoting controlled dehydration and formation of thermally stable, highly cross-linked char layers that protect the material against further pyrolysis and thermal degradation. Consequently, the higher residual mass observed for the immobilized systems likely results from the combined contribution of carbonized protein and char-protected PA6.

The TGA results confirm successful enzyme immobilization through the appearance of additional degradation events above 270 °C associated with protein decomposition. Pectinase immobilization significantly alters the TGA thermal degradation behavior of PA6 MPs, leading to improved thermal stability and a more complex degradation profile that depends, to some extent, on the pH of the pectinase solution used during immobilization. Similar improvements in thermal stability after immobilization of proteins or enzymes onto polyamide MPs have also been reported for systems containing bovine serum albumin [32] and laccase [33]. As CD analysis showed pH-dependent conformational changes in the enzyme structure, these changes probably also favor thermostability.

### 2.2. Determination of Catalytic Activity and Related Parameters

**Figure 4:**
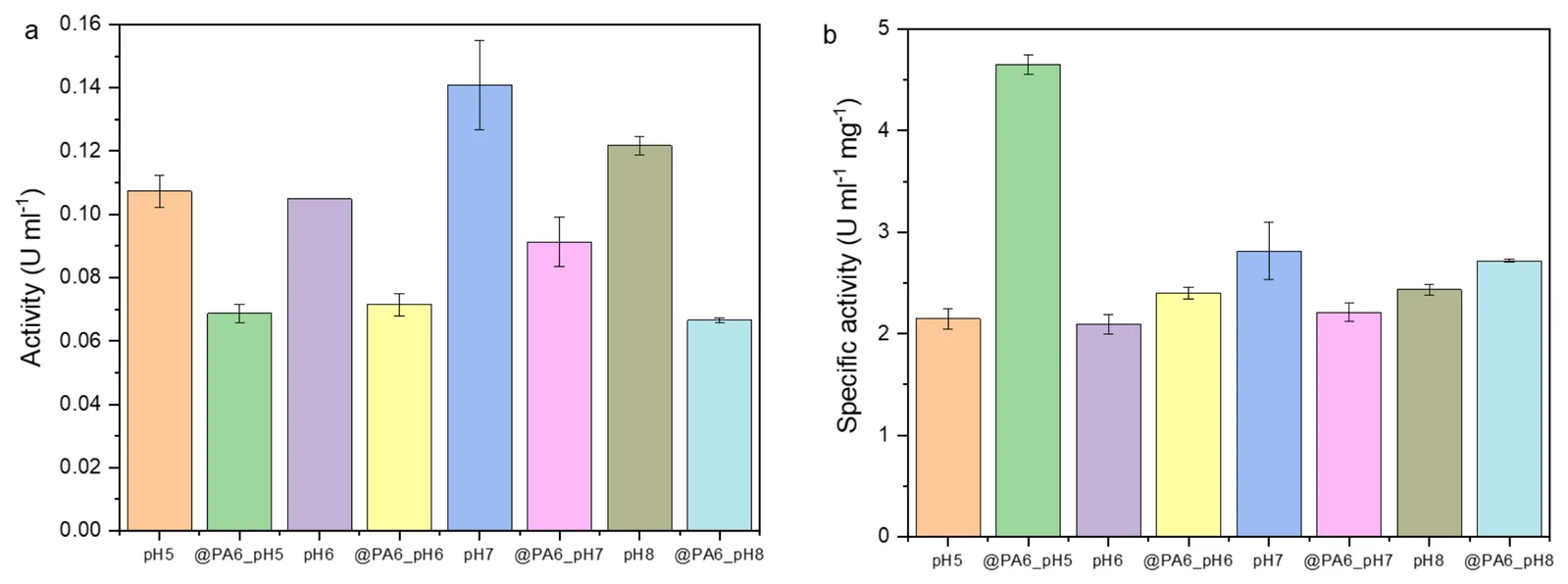
Studies of the activity of free pectinase solution and its immobilized complexes: (**a**) activity in units per milliliter (U/mL), where 1 U is defined as the amount of enzyme required to release 1 μmol of galacturonic acid per minute under the assay conditions, and (**b**) specific activity, representing the activity units per milligram of protein (U mL^−1^ mg^−1^). Standard deviation values were obtained based on two independent assays for each sample. The activity values of the free lyophilized pectinase (PeL) at pH 5–8 are designated as pH5–pH8; those of the corresponding immobilized complexes are designated as @PA6_pH5–@PA6_pH8.

The DNS method was used for the determination of the activity of free and immobilized PeL, as described in detail in Section 3.2.4. All activity assays were performed under identical conditions (pectin as the substrate in the citrate buffer (50 mM, pH 3.8, at 50 °C)), allowing direct comparison between free and immobilized systems. The results of pectinase activity and specific activity are presented in Figure 4.

The PeL and PeL@PA6 samples obtained at different immobilization pH values are presented together for comparison (Figure 4a). The activity of PeL_pH 7 is the highest, followed by PeL_pH8. The activities of PeL_pH5 and PeL_pH6 are similar. The immobilized complex PeL@PA6_pH7 exhibits the highest activity, while the remaining complexes show similar activities, slightly lower than those of the corresponding free enzymes.

When the activity values are normalized by the protein content (Figure 4b), the PeL@PA6_pH5 complex exhibits a specific activity approximately twice as high as that of the corresponding free enzyme (PeL_pH5). The complexes PeL@PA6_pH6 and PeL@PA6_pH7 also display specific activities that are statistically higher than those of their respective free enzyme samples. Only at pH 7 is the specific activity of the free PeL slightly higher than that of the immobilized complex. These results indicate that, in most cases, the fraction of PeL attached to the PA6 support remains highly active after immobilization. 

In addition to IY (Equation (1)) and activity (A, Equation (2)), two more important parameters related to enzyme immobilization were determined, namely: immobilization efficiency (IE, %) (Equation (3)) and activity recovery (AR, %) (Equation (4), Section Determination of Immobilization Efficiency and the Activity Recovery).

**Figure 5:**
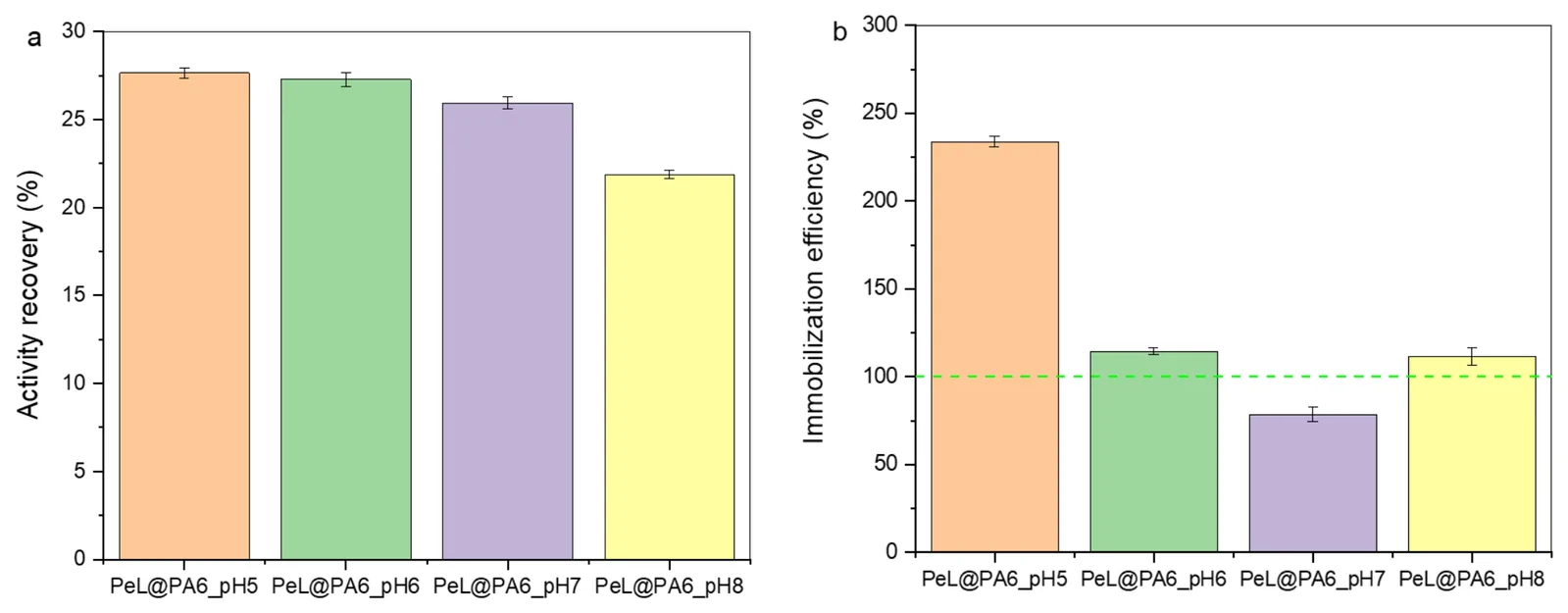
Studies of the activity recovery (**a**) and efficiency of immobilized pectinase (**b**). Standard deviations were calculated from two independent trials. The dashed line in (**b**) indicates the level of IE = 100%.

The evolution of AR and IE as a function of pH during immobilization on PA6 MPs are shown in Figure 5. Table 3 summarizes the immobilization parameters of all PeL@PA6 complexes at pH values between 5 and 8. The activity results in Figure 4 and Figure 5, and the consolidated numerical data in Table 3 demonstrate a clear dependence of the immobilization behavior and catalytic performance of the resulting PeL@PA6 conjugates on the pH of the enzyme solution.

The IY increases from 11.8% at pH 5 to a maximum of 33.0% at pH 7, followed by a decrease to 19.6% at pH 8. Evidently, enzyme adsorption immobilization onto the PA6 MPs becomes more effective as the pH approaches neutral conditions, perhaps due to a favorable balance between electrostatic interactions, hydrogen bonding, and hydrophobic contacts at the PeL/PA6 MP interface. At higher pH values, however, increased electrostatic repulsion between protein molecules and the MP surface (both negatively charged) may partially suppress adsorption, decreasing the IY observed at pH 8.

The catalytic properties of the immobilized systems show a different trend. The IE reaches its highest value at pH 5 (233.8%), which indicates hyperactivation. This effect can arise from several factors, including favorable enzyme orientation on the support surface, improved accessibility of the catalytic cleft, or stabilization of catalytically competent conformations of the enzyme upon PA6 support.

At pH 6 and pH 8, the immobilization efficiency remains slightly above 100% (114.6% and 111.7%, respectively), suggesting that the immobilization process still provides some stabilization or activation of the enzyme. In contrast, the conjugate prepared at pH 7 exhibits the lowest IE value (78.6%). Interestingly, this sample corresponds to the highest immobilization yield (see also Figure 1b). This indicates that a considerable fraction of the enzyme immobilized at pH7 may adopt less favorable orientations or experience steric constraints partially limiting catalytic accessibility. Such effects are typical for systems with higher enzyme loading, where crowding on the support surface or within pores increases steric and diffusional constraints, lowering the fraction of catalytically accessible enzyme molecules [34].

The AR values remain relatively similar for the conjugates obtained at pH 5–7 (25.9–27.6%), while a somewhat lower value is observed at pH 8 (21.9%). These moderate AR values indicate that only part of the initial enzymatic activity is retained after immobilization, which is common for adsorption-based immobilization systems [35]. 

Overall, all these results reveal a clear trade-off between immobilization yield and catalytic efficiency: although pH 7 provides the highest PeL loading, the most catalytically efficient conjugate is obtained at pH 5, where the lowest amount of immobilized enzyme exhibits clear hyperactivation.

### 2.3. Influence of Glucose and Ethanol on Enzyme Activity

The literature shows that the effectiveness of pectinases differs between activity assays and clarification applications, with higher apparent effectiveness generally observed in standardized activity assays [36]. This can be explained by the relatively high concentrations of sugars (mainly glucose and fructose) and increasing levels of ethanol during fermentation of must [37,38,39]. 

**Figure 6:**
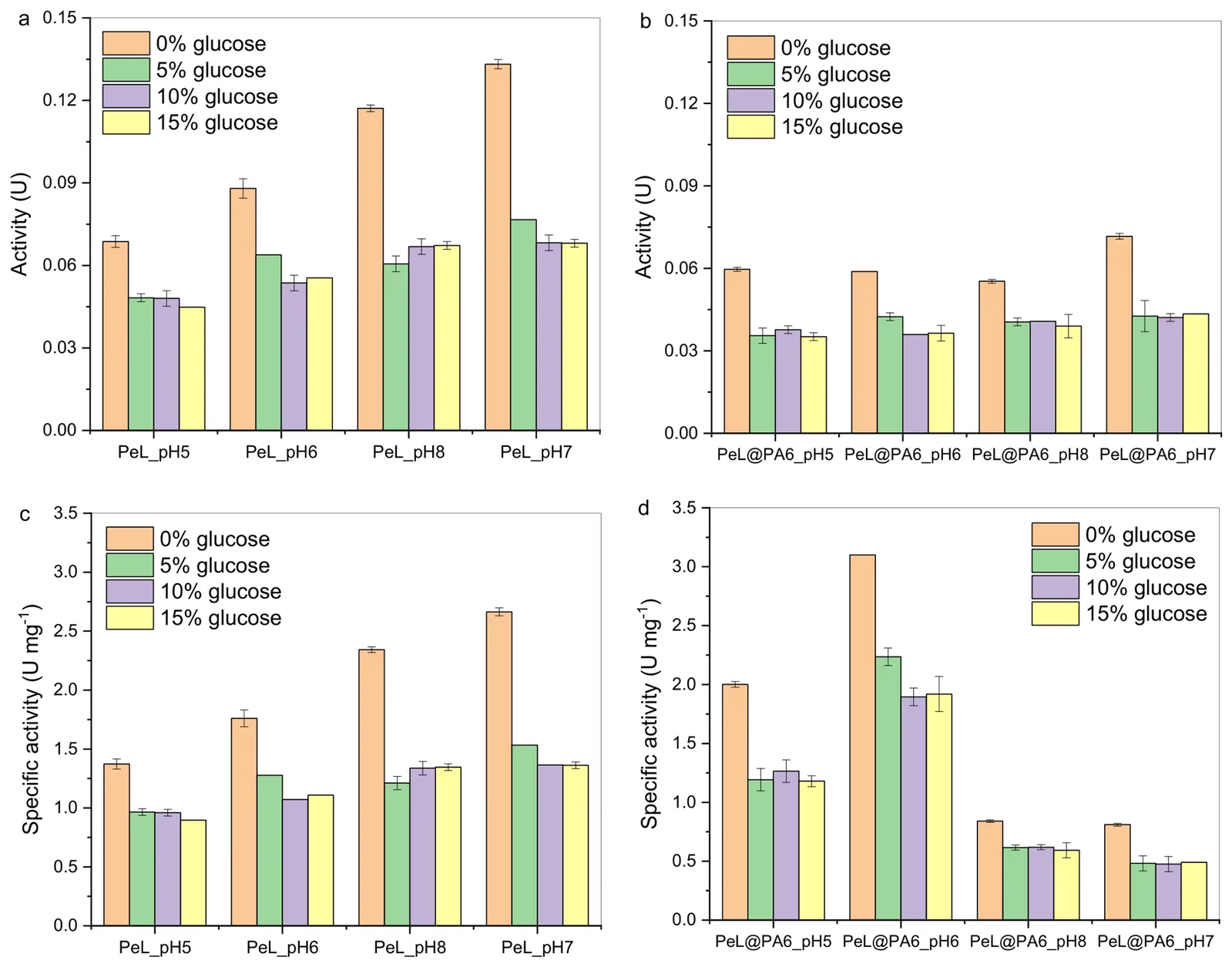
Effect of glucose concentration on enzymatic activity. (**a**) Free enzyme absolute activity; (**b**) immobilized enzyme absolute activity; (**c**) free enzyme specific activity; and (**d**) immobilized enzyme specific activity.

**Figure 7:**
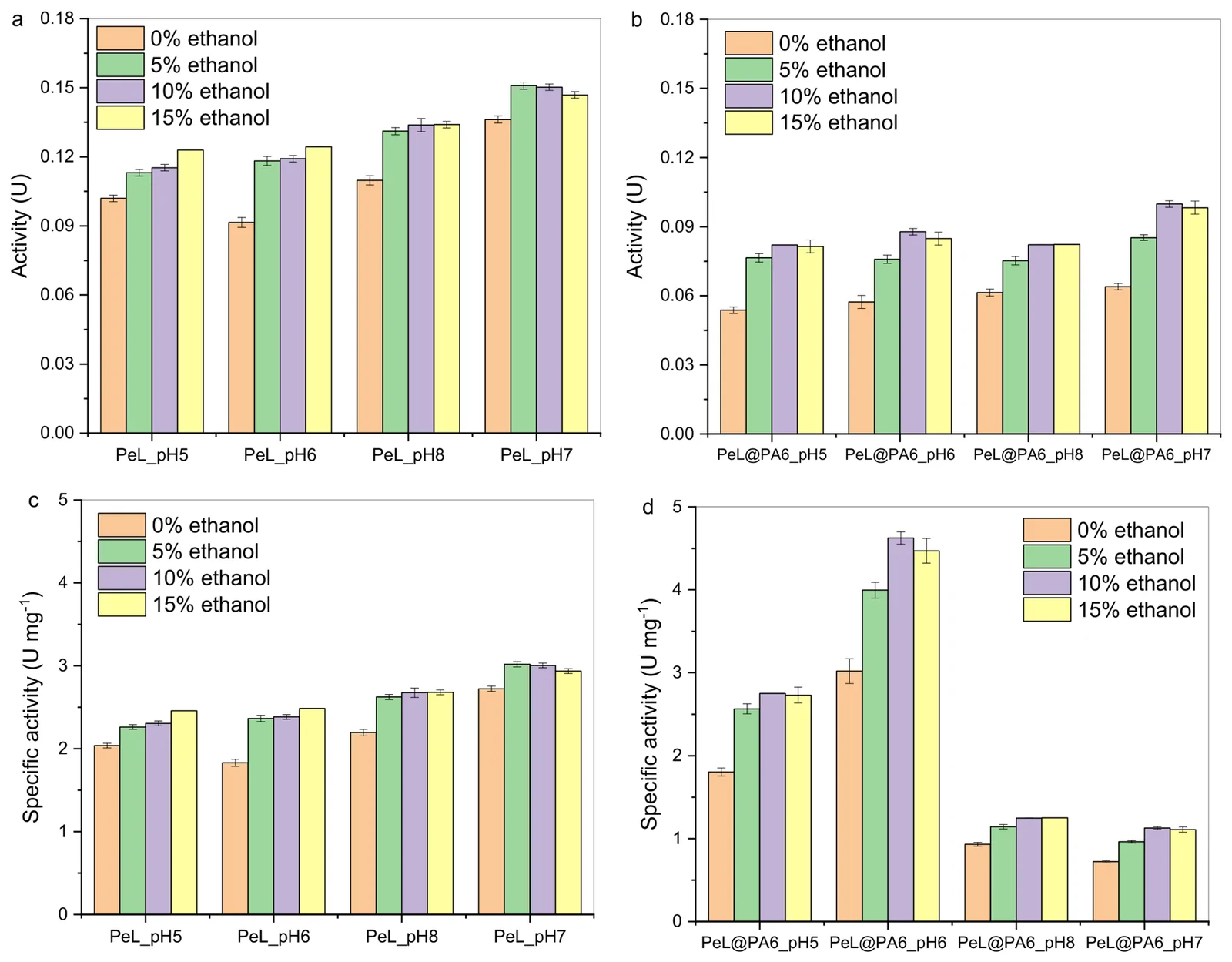
Effect of ethanol content on enzymatic activity. (**a**) Free enzyme absolute activity; (**b**) immobilized enzyme absolute activity; (**c**) free enzyme specific activity; and (**d**) immobilized enzyme specific activity.

To simulate conditions similar to those used in must clarification, PeL activity was evaluated in the presence of 5–15% (*w*/*v*) glucose and 5–15% (*v*/*v*) ethanol, as described in Section 3.2.5 and Section 3.2.6. The results of activity and specific activity are presented in Figure 6 and Figure 7.

As shown in Figure 6, glucose exerts a clear inhibitory effect on pectinase activity in both the free enzyme (Figure 6a,c) and the immobilized systems (Figure 6b,d), confirming previous observations reported by Radoi et al. [40]. In the absence of glucose, the activities of all samples (PeL and PeL@PA6) reach their maximum at pH 6–7 (Figure 6a,b). Upon addition of 5% *w*/*v* glucose, the activity decreases by approximately 20–30% for all systems. Further increasing the glucose concentration to 10–15% results only in minor fluctuations of the measured activities, remaining within the limits of experimental error. When the activities are normalized by protein content (Figure 6c,d), the dependence remains basically the same. 

The inhibitory effect of glucose can be attributed to changes in the microenvironment of the enzyme and possible conformational adjustments that reduce the accessibility of the catalytic cleft to the pectin substrate. High concentrations of glucose may also modify the hydration structure of the solution and increase medium viscosity, both of which can hinder substrate diffusion toward the active site. The absence of a strong additional decrease in activity between 10 and 15% glucose suggests that the enzyme approaches a limiting functional state under these conditions. 

Overall, these observations are consistent with a partial non-competitive inhibition mechanism, in which glucose does not compete directly with the substrate for the catalytic site but reduces the apparent catalytic capacity of the enzyme. 

The effect of added ethanol on the activity of free PeL (Figure 7a,b) and PeL@PA6 conjugates (Figure 6c,d) did not produce the inhibitory behavior commonly reported for organic solvents acting on enzymatic proteins [37,39]. In contrast, in the present study, the addition of ethanol (5–15% *v*/*v*) led to an increase in the catalytic activity (absolute and specific) of both free and immobilized pectinase. The pectinase activity displayed a non-linear dependence on ethanol concentration. At 5% ethanol, a significant increase in activity was observed (approximately 10–45% higher than the control without ethanol), suggesting a solvent-induced activation effect. At higher ethanol concentrations (10 and 15%), the activity decreased compared with that at 5%, although it remained above the control level. Such activation of pectinolytic activity in the presence of ethanol has already been reported in the literature. For example, at 15% ethanol (*v*/*v*), the pectinolytic activity of *Aureobasidium pullulans* GM-PN-22 increased by 24%, while in *Saccharomyces cerevisiae* B-17 an increase of 99% was observed [37].

In summary, the results indicate that glucose exerts an inhibitory effect on the enzymatic activity of lyophilized pectinase, both in the free and immobilized forms, whereas ethanol promotes pectin degradation within the studied concentration range. This behavior demonstrates that the enzyme exhibits good tolerance to ethanol. Consequently, the enzyme, whether free or non-covalently immobilized on PA6 microparticles, may operate more efficiently under the conditions typical of wine clarification than in the earlier stages of must processing. Conversely, when clarifying juices, where we have an increased glucose content, we will expect the opposite effect.

### 2.4. Kinetic Parameters

Kinetic studies are essential to evaluate the practical feasibility of immobilized enzymes in industrial bioprocesses. The kinetic parameters $V_{max}$ (i.e., the maximum reaction rate attained at substrate saturation) and $K_{m}$ (the apparent Michaelis constant reflecting the effective affinity of the enzyme for the substrate) of free and immobilized pectinases were determined by measuring the initial reaction rates for each enzymatic system using pectin concentrations ranging from 0.045 to 3.15 mg mL^−1^ in citrate buffer (50 mM) at constant pH (3.8) and temperature (50 °C), as described in Section 3.2.7. 

**Figure 8:**
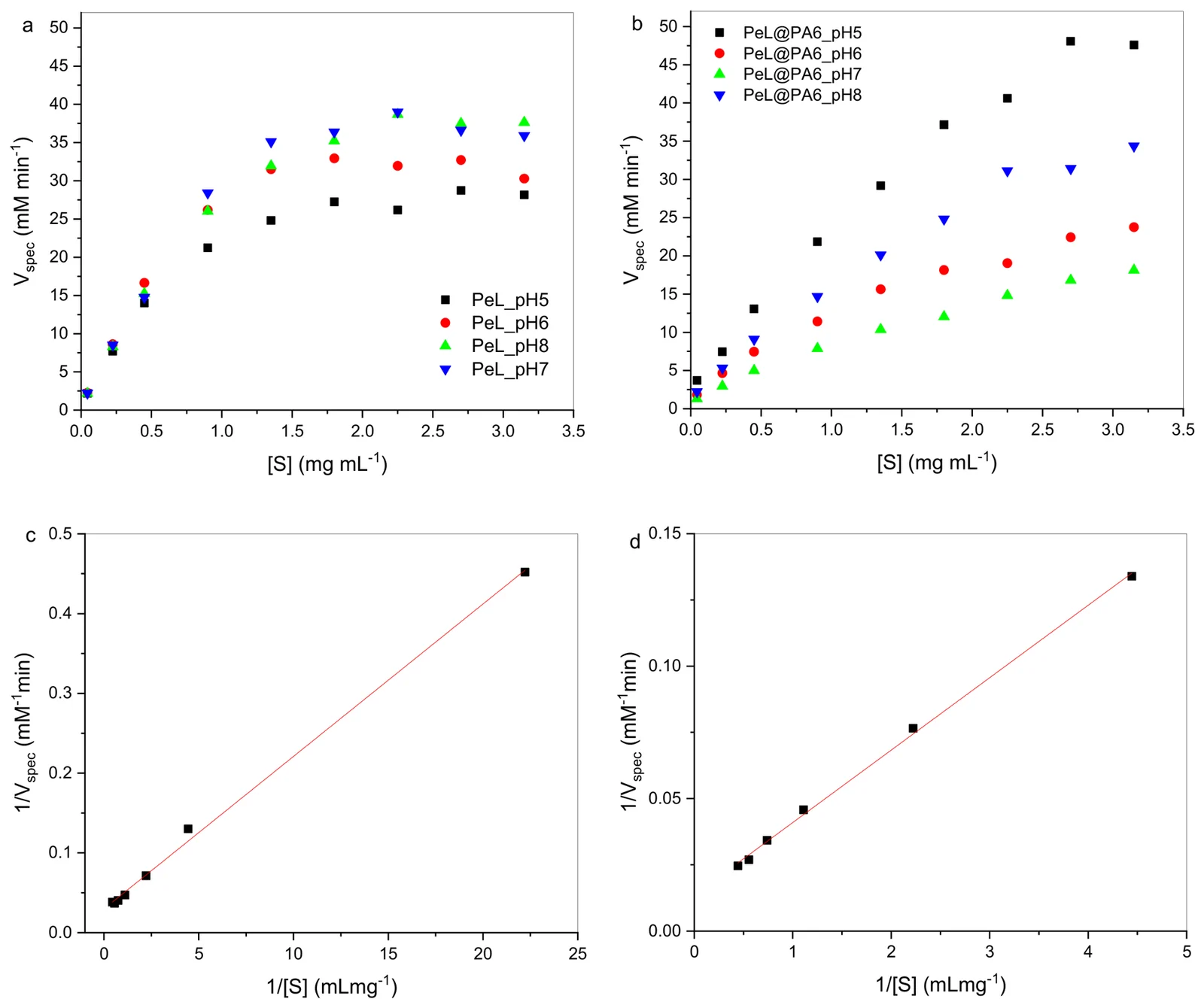
Selected experimental kinetic curves of free (**a**) and immobilized pectinases (**b**). Selected experimental Lineweaver–Burke plots of PeL_pH5 (**c**) and PeL@PA6_pH5 (**d**).

Figure 8a,b illustrates the kinetic analysis performed by plotting the specific activity per unit of protein $\left(V_{spec}\right)$ as a function of substrate concentration (S) following the Michaelis–Menten model. Figure 8c,d illustrates the Lineweaver–Burk double-reciprocal linearization.

The kinetic curves presented in Figure 8a for the free PeL at different pH values clearly demonstrate that, unlike the Viazym™ enological pectinase previously investigated [18,19], the lyophilized pectinase PeL follows classical Michaelis–Menten kinetics, showing a well-defined saturation plateau at substrate concentrations above 2 mg mL^−1^. In contrast, for the immobilized enzyme systems (Figure 8b), complete saturation was not reached within the substrate concentration range studied, with $\left(V_{spec}\right)$ still increasing slightly around substrate concentrations of 3.0–3.15 mg mL^−1^.

Therefore, the kinetic parameters $V_{max}$ and $K_{m}$ were estimated by nonlinear regression using the hyperbolic Michaelis–Menten equation (Equation (5), Section 3.2.7) and compared to the same parameters obtained using the Lineweaver–Burk double-reciprocal linearization (Equation (6)) presented in Figure 8c,d. The kinetic parameters obtained by nonlinear Michaelis–Menten regression, together with those calculated by the Lineweaver–Burk linearization for comparison, are summarized in Table 4. For discussion, the kinetic parameters obtained from nonlinear Michaelis–Menten fitting were prioritized due to their higher statistical reliability. As noted by Robinson [41], reciprocal linearization methods amplify experimental errors at low substrate concentrations and distort the statistical weighting of the data, which can lead to biased estimates of kinetic parameters, particularly $K_{m}$.

The results summarized in Table 4 show that immobilization at pH 5 and pH 8 produced enzyme–support complexes exhibiting higher $V_{max}$ values than the corresponding free enzymes. In particular, at pH 5, the immobilized enzyme displays a $V_{max}$ value of 93.061 mM min^−1^ mg^−1^, which is more than twice that of the free enzyme (35.327 mM min^−1^ mg^−1^), corresponding to an efficiency of approximately 223% and indicating a clear hyperactivation effect. In contrast, the complexes obtained at pH 6 and pH 7 exhibit $V_{max}$ values comparable to those of the corresponding free enzymes.

For all samples studied, however, a substantial increase in $K_{m}$ was observed. For example, at pH 5, the $K_{m}$ value increases from 0.654 (PeL) to 2.814 mg mL^−1^ after immobilization (PeL@PA6_pH5). Such increases in $K_{m}$ are frequently reported for immobilized enzyme systems and are generally attributed to mass-transfer limitations and diffusional restrictions rather than intrinsic changes in the enzyme–substrate interaction. Although the PA6 microparticles possess a highly porous morphology and large internal surface area, as demonstrated previously by SEM analyses [18,19], the diffusion of pectin molecules through the pore network toward the immobilized enzyme molecules may still be partially hindered. As a result, the effective substrate concentration at the catalytic sites decreases, leading to an increased apparent $K_{m}$. In other words, there is a weaker affinity of the enzyme for its substrate, but once the active site is reached, a rapid reaction follows, being much faster than that with the free enzyme. This is evidenced by the high values of the specific activity of $K_{m}$. 

**Figure 9:**
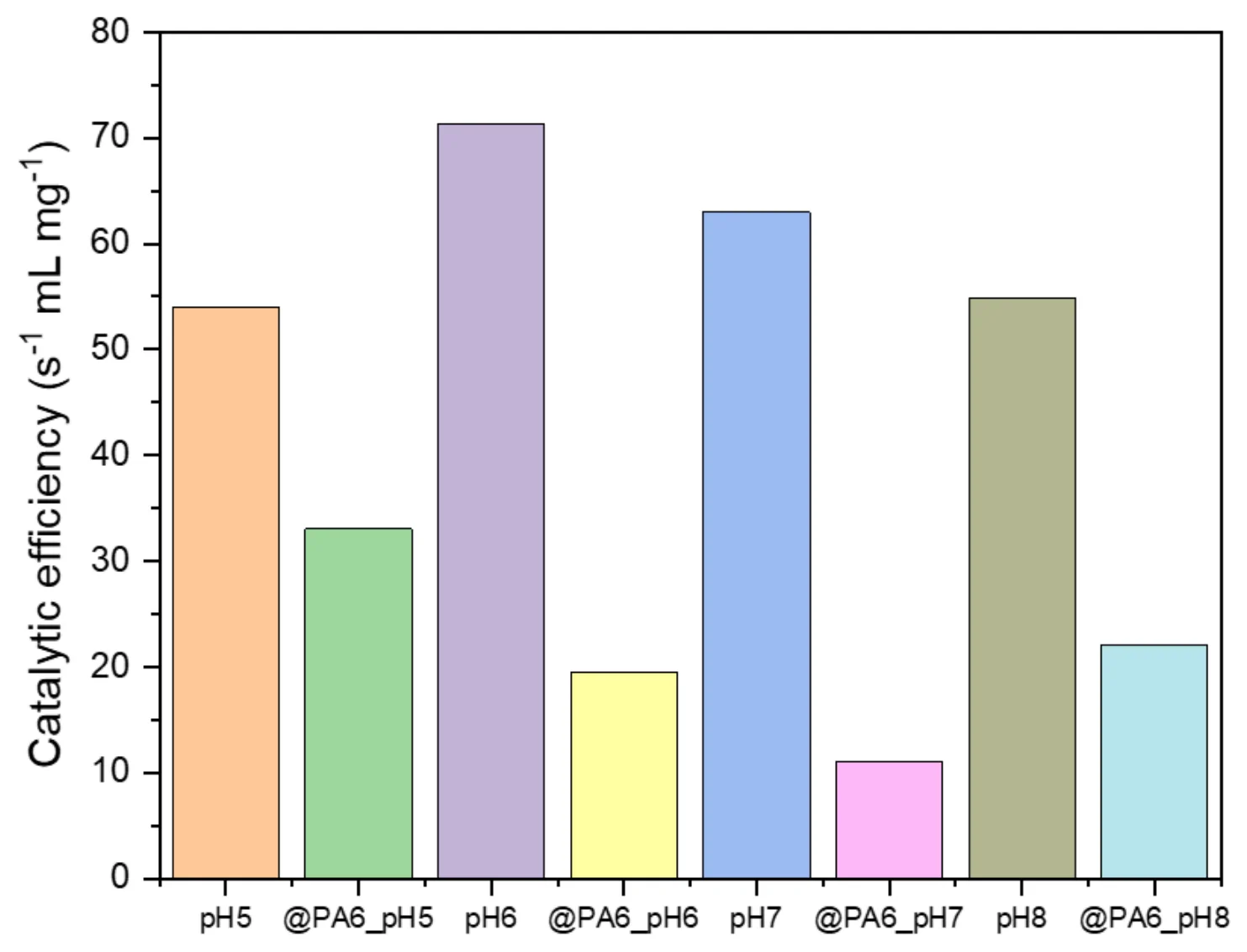
Catalytic efficiency (CE) of the free and immobilized pectinases calculated from the kinetic parameters obtained by nonlinear Michaelis–Menten fitting. The CE values of the free lyophilized pectinase (PeL) at pH 5–8 are designated as pH5–pH8; those of the corresponding immobilized complexes are designated as @PA6_pH5–@PA6_pH8.

The overall catalytic performance of the systems was evaluated through the catalytic efficiency (CE), defined as the ratio $V_{max}/K_{m}$, as presented in Figure 9. Apparently, the *CE* values of free PeL are higher than those of the immobilized systems. Although $V_{max}$ increases significantly in some cases, particularly for the conjugate prepared at pH 5, the concurrent increase in $K_{m}$ ultimately reduces the overall catalytic efficiency of the immobilized enzyme. This behavior can be attributed primarily to limited substrate accessibility, as a fraction of the immobilized enzyme molecules may reside within internal pores of the PA6 MPs where substrate diffusion is restricted. Additionally, adsorption of the enzyme onto the polyamide surface may impose steric constraints that partially limit substrate access to the catalytic cleft, further contributing to increased apparent $K_{m}$ values and lower CE.

### 2.5. Application of PeL@PA6 Complexes for Rosé and White Wine Must Clarification

To evaluate the potential industrial applicability of the immobilized enzymes, they were applied to the clarification of rosé and white must. In addition to differences in their production processes, these musts differ in composition, particularly regarding polysaccharide and monosaccharide content, which can influence enzyme performance during clarification [42,43]. 

**Figure 10:**
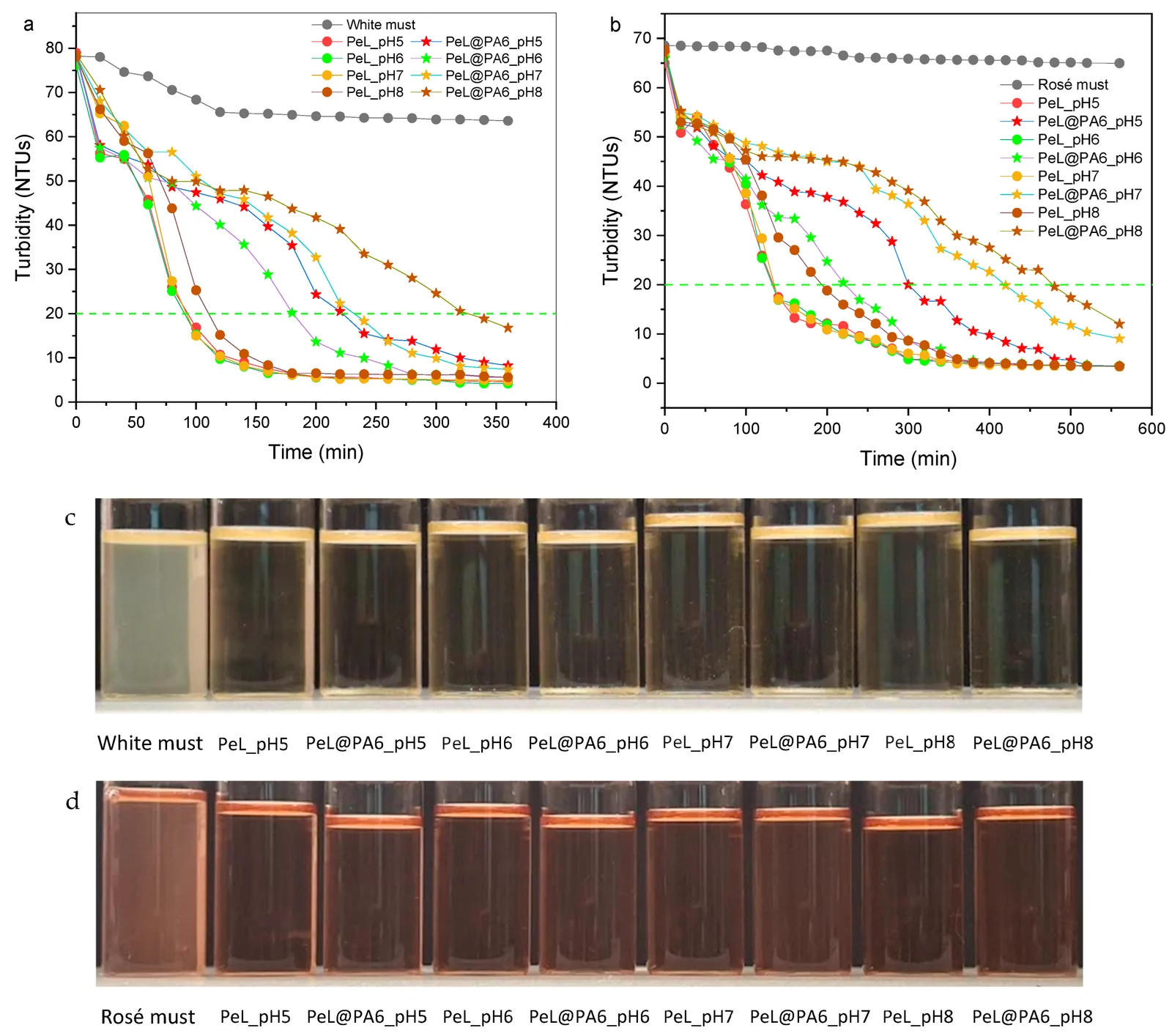
Clarification time dependence for white (**a**) and rosé (**b**) must with free and immobilized pectinase preparation. The dots are for the free enzymes and immobilized various pH values, and the stars—for the respective PeL@PA6 immobilized complexes. Visual aspect of the clarified samples for all catalytic systems applied in white (**c**) and rosé (**d**) musts before and at the end of the process.

White wine and rosé musts, with starting pHs of 3.19 and 3.44, respectively, containing 2.0 mg mL^−1^ of pectin, were clarified using 180 µL of pectinase solution containing 0.1 mg of free pectinase (pH 5–8) and the corresponding amount of immobilized enzyme (prepared at pH 5–8) at room temperature (22 °C ± 2 °C), as described in Section 3.2.8. The results of clarification studies and the visual aspect of the must after and before clarification are presented in Figure 10. The graphs shown in Figure 10a,b reveal distinct clarification behaviors and clarification times (defined as turbidity values below 20 NTU). 

In untreated white must (Figure 10a, black dots), the initial turbidity was around 80 NTU and stabilized close to 65 NTU after 120 min with no further changes. The decrease in turbidity started immediately upon the addition of PeL (free or immobilized), and after 20 min all systems exhibited turbidity values of approximately 55 NTU, i.e., lower than the stabilization level of the untreated white wine must. Both free and immobilized enzyme samples at pH 5 and pH 6 showed the fastest clarification, whereas those at pH 8 were slightly slower. After 100 min, all free enzyme samples achieved turbidity below 20 NTU. The same turbidity for the white wine must was reached by PeL@PA6_pH6 after 200 min, while the complexes immobilized at pH 5, pH 7, and pH 8 required 240, 240, and 340 min, respectively, to reach the desired turbidity. Visual differences in clarified white must samples are shown in Figure 10c. 

In the case of rosé must (Figure 10b,d), the turbidity of the untreated sample remained nearly constant at approximately 70 NTU throughout the whole clarification period. Due to the higher concentration of cell wall-derived pectic components [42], this turbidity is slightly higher than that observed for white must. The enzymatic treatments followed a similar trend to that observed for white must, with free enzymes clarifying the must more rapidly. However, for rosé must, clarification required approximately 40 additional minutes for free PeL immobilized at pH 5, pH 6, and pH 7, and more than twice as long for the enzyme in the PeL_pH8 system. 

Among the immobilized systems, the PeL@PA6_pH 6 complex was the fastest, reaching turbidity values below 20 NTU after 240 min (4 h), followed by the PeL@PA6_pH 5 complex, which required 320 min. The slowest system was the complex immobilized at pH 8, which required 480 min (8 h) to achieve the target turbidity. 

These results are consistent with kinetic studies, as complexes with higher $K_{m}$ values exhibited slower clarification rates, reflecting lower apparent substrate affinity. The results are also in agreement with the catalytic efficiency data, as the free enzyme consistently displayed faster clarification performance than the immobilized systems.

Notably, tests for residual pectin (Section 3.2.9) showed no presence of pectin in the white wine and rosé must samples with turbidity levels equal to or below 20 NTU. Moreover, the pH values of the two types of must before and after clarification remained almost unchanged, about 3.12–3.15 and 3.38–3.44, respectively. These data are presented in the Supplementary Materials (Figure S4 and Table S1) and suggest that the enzymatic treatment with free and immobilized PeL produced pectin-free musts with natural acid-base balance. 

### 2.6. Color Analysis of Musts

The color of the must/wine is a primary attribute determining consumer preference and a critical quality parameter subject to rigorous control. It is influenced by both the must’s pH and the action of enzymes during the winemaking process [44,45].

The color control of the must after clarification with free and immobilized enzymes was performed by acquiring UV/VIS absorption spectra, as described in Section 3.2.10. The color of the must was evaluated at three specific wavelengths: 420 nm (yellow component), 520 nm (red component), and 620 nm (blue component) [46]. 

**Figure 11:**
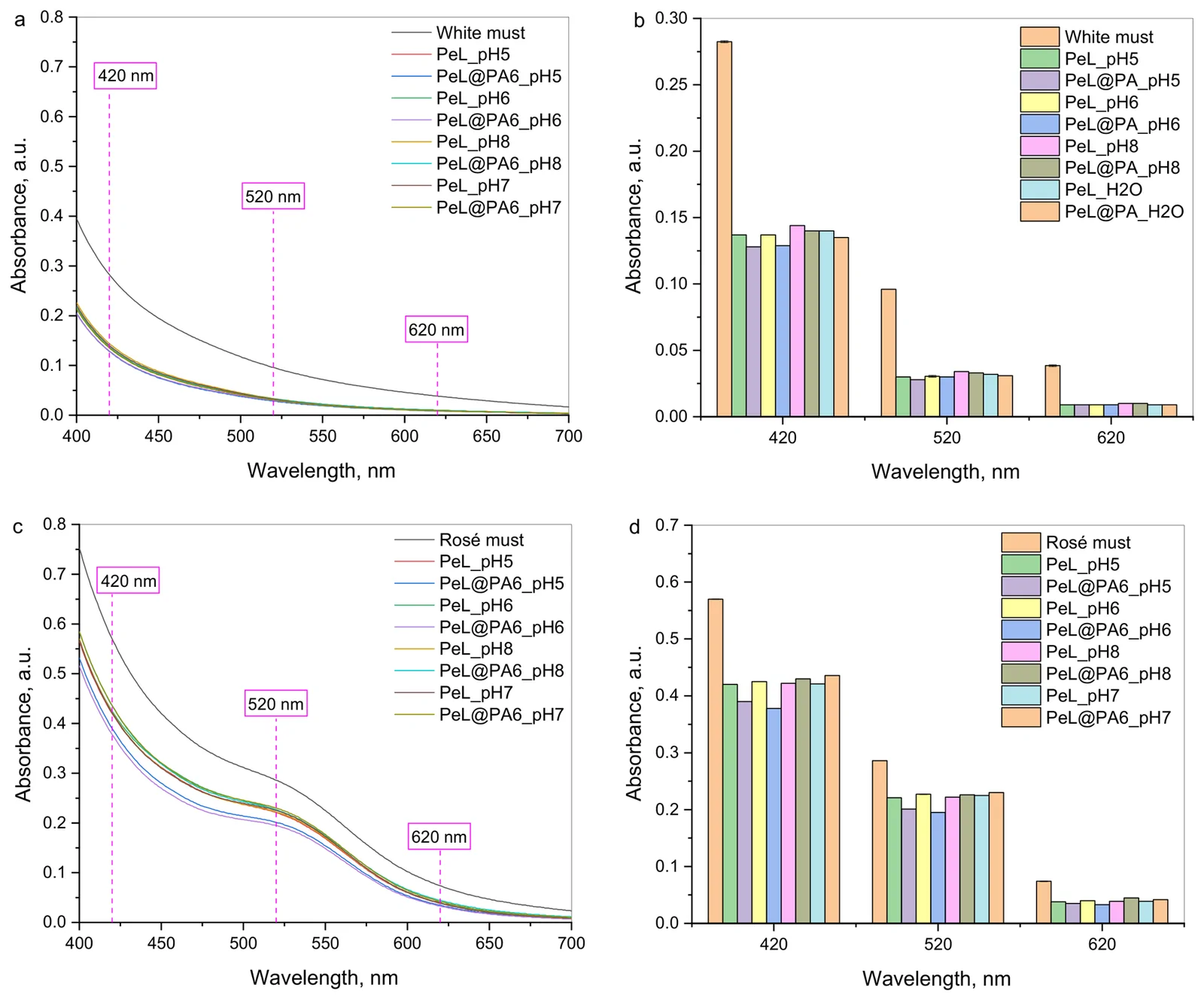
Analysis of must color changes after clarification. (**a**,**c**) Comparison of UV/VIS absorption spectra in three characteristic regions (420, 520, and 620 nm) for white and rosé must, respectively, and (**b**,**d**) comparison of numerical color parameters for white and rosé must, respectively.

The results of the color analysis are presented in Figure 11.

The UV/VIS spectra shown in Figure 11a,c, together with the bar graphs presented in Figure 11b,d, indicate that must treated with immobilized enzymes exhibits absorbance values at 420, 520, and 620 nm comparable to those obtained with the corresponding free enzymes. The use of porous microparticulate enzyme supports could potentially affect must color due to selective adsorption of certain compounds onto the microparticles. However, the results demonstrate that the PA6 MPs employed in this study do not induce undesirable color changes in the must.

For a more detailed evaluation of must color and composition before and after clarification, several additional parameters were determined, including color intensity (I), which provides an objective quantitative assessment of must/wine color; the relative contribution of each absorption component to the overall color intensity (% Yellow (Y), % Red (R), and % Blue (B)); tonality (T), which reflects the chromatic characteristics of the must; browning index (BI), associated with oxidation reactions that lead to brown coloration and changes in brightness; total polyphenolic index (TPI), which estimates the overall phenolic content; hydroxycinnamic acids (HCA); and flavanols (FLAV). All parameters were measured or calculated as described in Section 3.2.10. The results are summarized in Tables S2 and S3 of the Supplementary Materials.

Table S1 presents the color and compositional parameters of white wine must before and after clarification. The BI value of untreated must is higher than that of treated samples, suggesting that both free and immobilized pectinases are effective in limiting oxidation phenomena. The I of white must decreased significantly after clarification, from 0.417 to approximately 0.17, indicating a high degree of clarification. The increase in T reflects the removal of suspended solids and unstable pigments, resulting in a characteristic straw-yellow hue and improved color stability. The TPI, HCA, and FLAV values remained relatively constant after treatment, indicating that neither free nor immobilized enzymes promote the removal of important phenolic compounds.

Table S2 summarizes the corresponding results for rosé must. In this case, BI values were also lower after enzymatic treatment, indicating reduced oxidative effects. The I decreased from 0.93 in untreated must to values between 0.6 and 0.7 after clarification, suggesting partial removal of unstable colored polymers and improved color stabilization. Since rosé must contains anthocyanins, which may potentially be adsorbed by PA6 MP supports, the similarity in tonality values between musts treated with free and immobilized enzymes indicates that the PA6 support does not significantly adsorb these pigments. As observed for white must, the TPI, HCA, and FLAV values remained essentially unchanged, confirming that the polyamide 6 microparticles do not remove relevant phenolic fractions from rosé must.

In summary, the microparticulate polyamide supports used for non-covalent enzyme immobilization do not induce undesirable changes in the color or phenolic composition of either white or rosé must, demonstrating their suitability for enological clarification applications.

### 2.7. Reusability of Immobilized Pectinase

The possibility of reusing enzymes is an important advantage of enzyme immobilization [47]. In this work, reuse was evaluated both in must clarification and in enzymatic activity assays.

**Figure 12:**
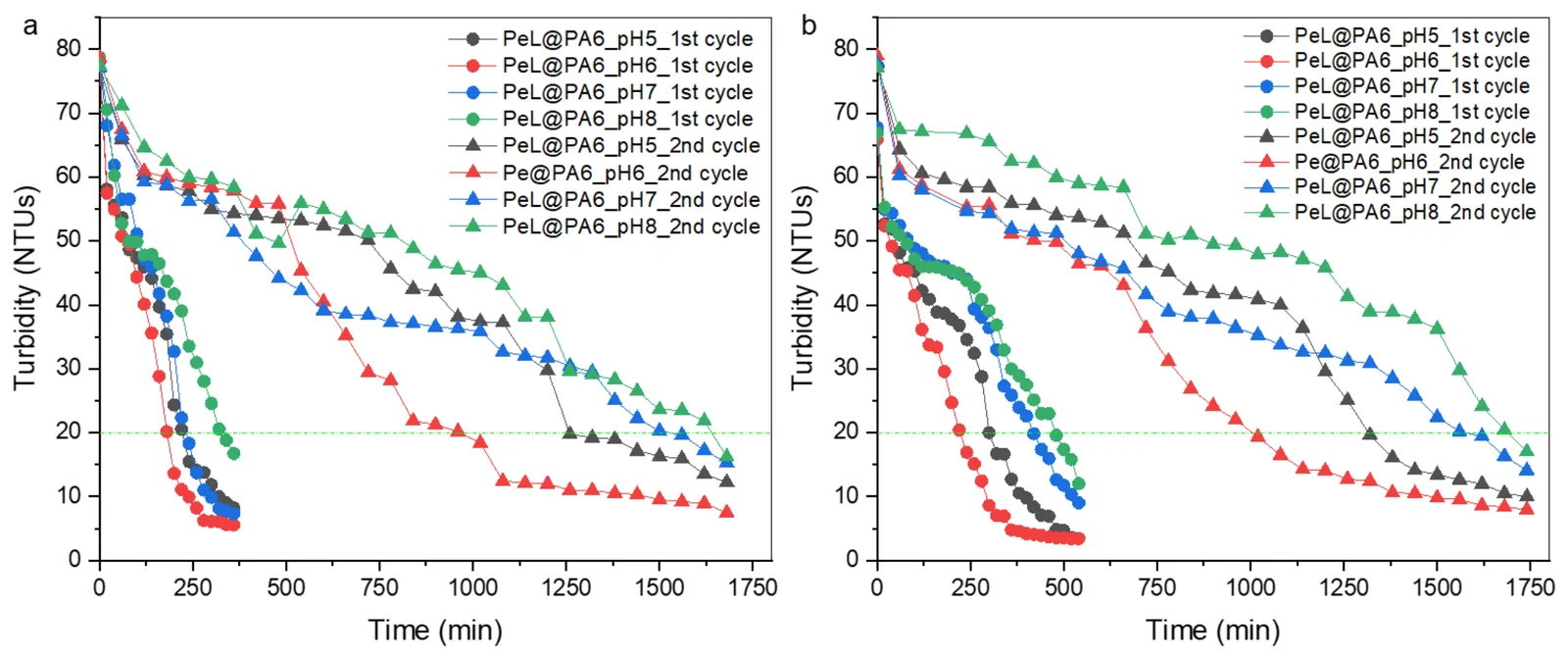
Reuse of immobilized enzyme in the clarification of white (**a**) and rosé (**b**) must.

The PeL@PA6 complexes were used in two consecutive clarification cycles, as described in Section 3.2.11, and the results are presented in Figure 12.

The complexes successfully reduced turbidity to values below 20 NTU (thus fully eliminating pectin) in both clarification cycles: in the first cycle, 6 h for white must (Figure 12a) and 9 h for rosé must (Figure 12b); in the second cycle, 28 h for white must (Figure 12a) and 29 h for rosé must (Figure 12b). PeL@PA6_pH6 was the fastest system in clarifying both musts, in good agreement with its lowest K_m_ value.

Despite the longer clarification time required during the second reuse cycle, all immobilized pectinase systems still achieved the target turbidity (<20 NTU), demonstrating that the biocatalysts remained effective for must clarification under the investigated conditions. The longer clarification time observed during the second cycle may be attributed to several factors, including partial enzyme deactivation, gradual enzyme desorption, and/or adsorption of hydrolysis byproducts onto the microparticle surface, which could hinder substrate diffusion and reduce access to the enzyme active sites. Since the relative contribution of these factors was not specifically investigated in the present work, no definitive mechanism can be proposed. Nevertheless, the results demonstrate that the immobilized biocatalysts can be successfully reused in two consecutive clarification cycles while maintaining clarification performance within the limits generally accepted for industrial wine production. Under industrial conditions, where clarification is typically performed without stirring or gas bubbling, processing times of up to 48 h are commonly accepted. 

**Figure 13:**
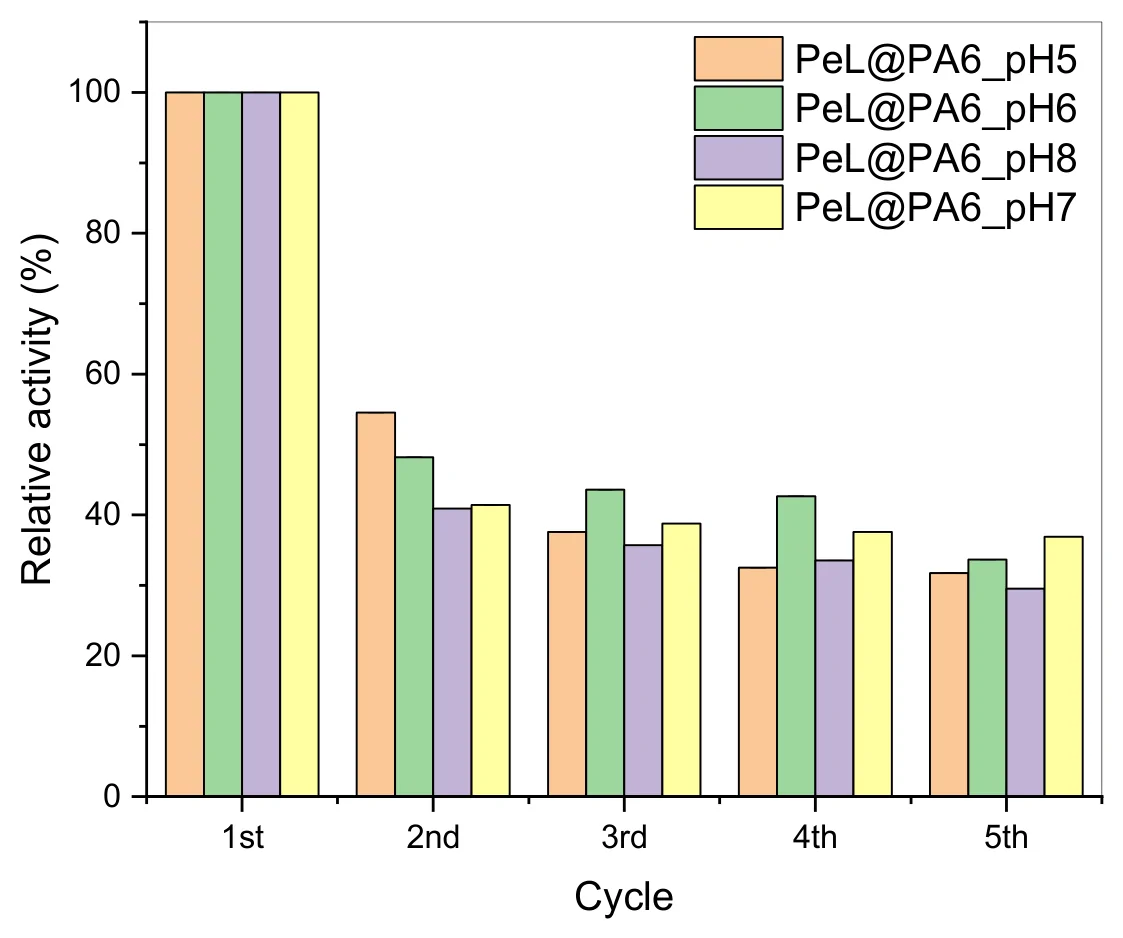
Reuse of immobilized enzymes over five activity test cycles. Two tests were performed for each sample, with standard deviations ranging from 0.01 to 0.03%, i.e., within the margin of the experimental error.

The complexes were also used for five consecutive cycles of enzymatic activity testing, using pectin as a substrate at pH 3.8 and 50 °C, as described in Section 3.2.12 of this work. The results of the enzymatic activity reuse tests are summarized in Figure 13.

After the first reuse cycle, the PeL@PA6_pH5 and PeL@PA6_pH6 systems retained 55–48% of their initial activity, while the remaining systems retained approximately 40%. In subsequent cycles, the activity remained between 30 and 40% of the initial activity. The best-performing system was PeL@PA6_pH7, which retained approximately 35% of its initial activity at the end of the fifth reuse cycle. Overall, the activity decreased significantly after the first cycle and then remained relatively stable up to the fifth reuse cycle. The loss of activity is likely due to partial enzyme desorption from the microparticles, leaving only the more strongly bound enzyme fraction. However, the stabilization of residual activity between the second and fifth cycles suggests a robust interaction between the enzyme and the microparticles, which prevents further leaching under the tested conditions.

From a practical standpoint, the immobilized systems can be reused in consecutive cycles of must clarification and enzymatic activity, demonstrating a clear advantage of immobilization and an industrial benefit by enabling enzyme reuse. The reuse results obtained in clarification and activity assays are not directly comparable, as must is a much more complex medium than the substrate solution used in activity tests, which explains the differences observed between the two reuse studies, the first one being more valuable for practical applications.

### 2.8. Storage Stability

**Figure 14:**
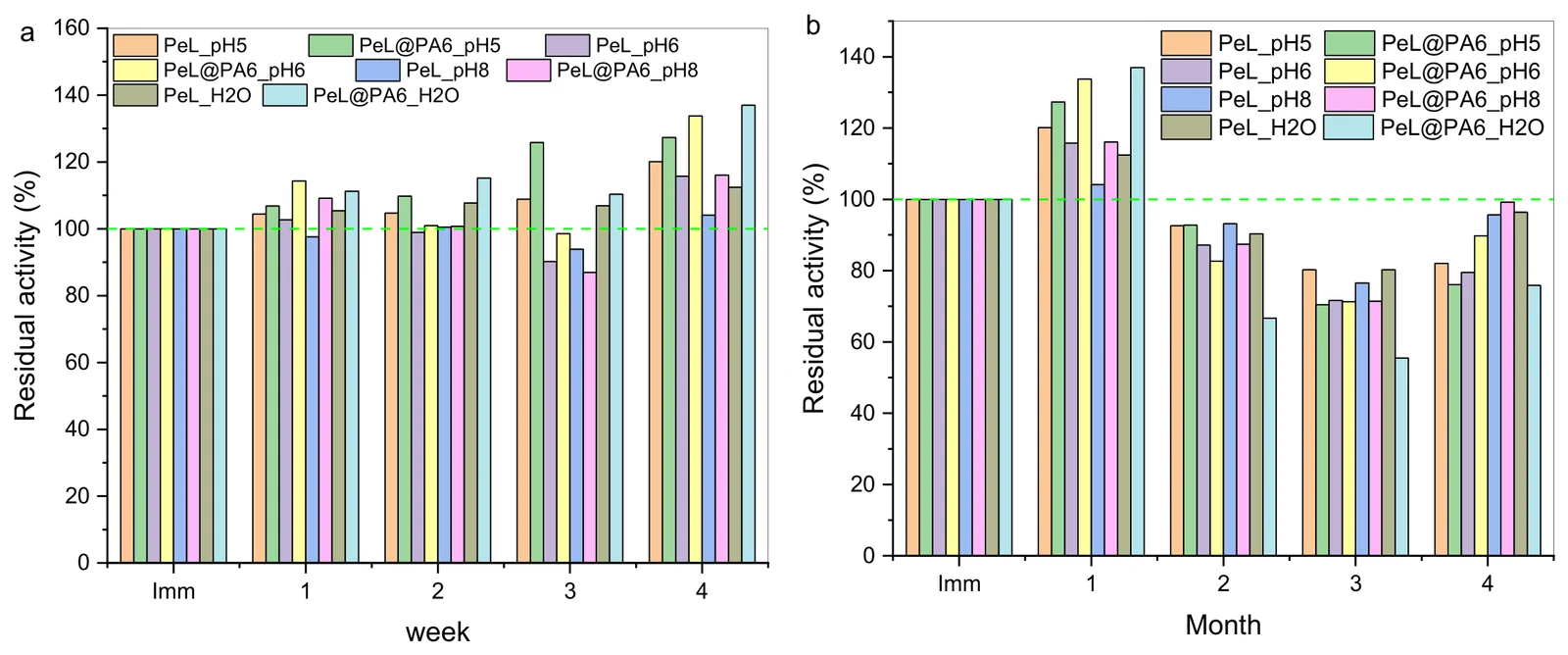
Storage stability profile at 4 °C of free and immobilized pectinases: (**a**) stability over 4 weeks and (**b**) stability over 4 months. The dashed green line corresponds to 100% activity. Standard deviation values were obtained from two independent assays for each sample and ranged between 0.02 and 0.05%, i.e., within the margin of the experimental error.

Stability during storage is one of the most important characteristics of enzymes, as it directly affects their applicability in industrial processes. Evaluating storage stability allows the assessment of biocatalyst performance under defined storage conditions. In this study, free and immobilized pectinases were stored at 4 °C, and their activities were periodically determined over four months using the DNS method as described in Section 3.2.13. Figure 14 shows the residual activity profiles of the free and immobilized enzymes during the evaluated period.

The initial activity was considered 100%. During the first month of evaluation (Figure 14a), enzymatic activities increased in almost all cases. In the third week, PeL at pH 6 and pH 8, as well as PeL@PA6 complexes at pH 6 and pH 8, exhibited activities below 100%. However, these values increased again in the fourth week. Consequently, after four weeks of storage, both the free enzyme and the immobilized complexes showed activities higher than their initial values. In all cases, immobilized enzymes exhibited higher activity than their respective free enzymes, with PeL@PA6_pH7 showing the highest activity.

When storage stability was evaluated over a period of four months (Figure 14b), a different behavior was observed. In this case, all enzymatic activities were lower than their respective initial activities. Activity decreased during the first three months, followed by a slight increase in the fourth month of storage. Overall, the activity of the immobilized complexes was lower than that of the corresponding free enzyme, except for the PeL@PA6_pH6 and PeL@PA6_pH8 systems, which exhibited slightly higher activity than their respective free enzymes. This trend suggests that while the PA6 support provides initial stabilization, long-term storage may lead to some enzyme leaching or slow protein denaturation within the porous structure of the microparticles.

These results suggest that both free and immobilized biocatalysts exhibit hyperactivation, as evidenced by residual activities higher than 100% after four weeks of storage. The hyperactivation of pectinases has been previously reported by Bustamante-Vargas et al., who observed a residual activity of 198% after 229 days for an immobilized pectinolytic system [40]. In the present study, this phenomenon may be associated with the presence of the polyamide support, which can provide external protection to the enzyme and create a favorable microenvironment. This microenvironment may enhance pectinolytic activity by protecting the enzyme from adverse temperature effects and by mimicking confinement and aggregation effects similar to those observed in living cells [41]. Furthermore, the gradual hydration of the polyamide matrix over time may improve the accessibility of the substrate to the enzyme’s active sites, while the synergistic action between the different enzymes in the commercial cocktail (e.g., pectin methyl esterases and polygalacturonases) may be optimized during the initial storage period, leading to the observed hyperactivation. Additionally, conformational rearrangements of the enzyme during storage may contribute to maintaining the enzyme in a more active state.

Summarizing, PeL@PA6 pectinolytic systems are recommended to be used within 2 to 3 weeks after immobilization in order to preserve their enhanced activity compared to free PeL. This storage behavior allows flexibility in enzymatic preparation, enabling them to be prepared and stored before application, a significant advantage for an industrial application. 

## 3. Materials and Methods

### 3.1. Materials

Lyophilized pectinase preparation (endo-polygalacturonase, EC 3.2.1.15; CAS 9032-75-1) from *Aspergillus niger* (Thermo Fisher Scientific Chemicals, Waltham, MA, USA) was used throughout this study. The enzyme was supplied as a white powder with a minimum specific activity of 20.0 U/mg (dry weight). One unit (U) was defined as the amount of enzyme that liberates 1.0 µmol of galacturonic acid per minute. The powder was stored at room temperature in a dry environment until further use.

Industrial rosé and white musts were kindly donated by Sogrape Vinhos (Avintes, Portugal). The musts were stored at −10 °C to prevent fermentation until further use.

The synthesis of the anionic PA6 MPs was performed via activated anionic ring-opening polymerization (AAROP) in solution following a previously described synthetic route [48]. The following materials were used in the synthesis: AP-NYLON^®^ ε-caprolactam monomer and the AAROP activator BRUGGOLEN^®^ C20P, both supplied by Brüggemann (Heilbronn, Germany), as well as the AAROP initiator Dilactamate^®^ supplied by Katchem (Prague, Czech Republic). The general reaction scheme of this polymerization process is illustrated in Figure S5 of the Supplementary Materials.

As described in detail elsewhere [21,48], the AAROP produces PA6 microparticles with purification yields of 65–72% (relative to the caprolactam monomer), a viscometric molecular weight M_v_ of 35,000–38,000 g mol^−1^, an equivalent circular diameter d_max_ of 25–45 µm, a roundness (d_max_/d_min_) of 1.1–1.3, and SEM-observed pore sizes of 350–600 nm. The specific surface area of the PA6 microparticles, determined by the BET method, ranges from 4.6 to 7.4 m^2^ g^−1^. The PA6 MPs exhibit a crystallinity index of 45% (WAXS), comprising predominantly α-PA6 (33%) and γ-PA6 (12%) crystalline phases. DSC measurements showed a melting temperature *T_m_* of 207–208 °C and a glass-transition temperature *T_g_* of 32–35 °C. 

All other chemical reagents, including pectin from citrus peel, solvents used in the AAROP synthesis and other experiments, and reagents used for buffer preparation, were of analytical grade and supplied by Sigma-Aldrich–Merck (Lisbon, Portugal). 

### 3.2. Methods

#### 3.2.1. Immobilization Method

The PeL from Thermo Fisher Scientific Chemicals was immobilized onto PA6 MPs by physical adsorption. Enzyme solutions with a concentration of 0.5 mg/mL were prepared in different media: sodium phosphate buffer (0.1 M) was used for experiments at pH 6–8, whereas citrate buffer (0.1 M) was employed at pH 5 because phosphate buffer does not provide sufficient buffering capacity in this pH range. For each immobilization condition, 5 mL of the enzyme solution was added to 100 mg of PA6 MPs in Eppendorf tubes. The resulting suspensions were incubated at room temperature (20 ± 2 °C) for 24 h under gentle orbital shaking to promote enzyme adsorption onto the polymer support. Accordingly, the initial enzyme concentrations of 0.5 mg mL^−1^ correspond to 25 mg PeL g^−1^ PA6 support.

After incubation, the suspensions were centrifuged at 5000 rpm for 5 min, and the supernatants were collected and stored at 4 °C for further analysis. The resulting PeL@PA6 complexes were washed twice with double-distilled water to remove non-adsorbed enzyme and then stored at 4 °C until further use.

#### 3.2.2. Characterization Methods 

The TGA measurements of the empty PA6 MPs and the PeL@PA6 complexes obtained by immobilization at pH values between 5 and 8 were carried out using a Q500 thermogravimetric analyzer (TA Instruments, New Castle, DE, USA). The samples were heated from 40 to 600 °C at a heating rate of 10 °C/min under a nitrogen atmosphere. The typical sample weights of the dried materials were in the range of 10–20 mg.

All conventional UV–VIS spectral measurements were performed using a Shimadzu UV-1900i double-beam spectrophotometer (Shimadzu Corporation, Kyoto, Japan) operating in photometric or spectral mode and equipped with quartz cuvettes with an optical path length of 1 cm.

The secondary structure of the free lyophilized PeL and the supernatants remaining after immobilization was studied by CD UV-VIS spectroscopy using a JASCO Corporation (Hachioji, Tokyo, Japan) spectropolarimeter model J-1500. The measurements were performed at room temperature (20 ± 2 °C) using a quartz cuvette with an optical path length of 1 mm. The baseline was recorded using the respective buffer solution. The spectra were acquired in the 260–185 nm range at a scan speed of 20 nm/min and a bandwidth of 1 nm. The final spectra represented the average of three consecutive scans for each sample. The CD data were analyzed using the DichroWeb online server [49].

The turbidity measurements were made using a METRIA M10 portable turbidimeter (Labbox Labware, Barcelona, Spain), performing up to 5 parallel measurements with every sample, according to an ISO standard [50].

#### 3.2.3. Determination of the Total Amount of Protein

After immobilization, the supernatants were analyzed by conventional UV/VIS spectroscopy to determine the residual protein content and calculate the total protein (TP) incorporated into the PA6 MPs, according to:

(1) $$TP=C_{0}-C_{S^{\prime }}$$

where $C_{0}$ is the initial protein content before immobilization, and $C_{S^{\prime }}$ is the protein content in the supernatant after the immobilization process. Protein concentration was determined by direct quantification of the maximum UV absorption peak at λ ≈ 277 nm. In this study TP is considered as immobilization yield, *IY* [%]. 

#### 3.2.4. Determination of PeL Activity and Related Parameters

##### Activity Assay

The activity of pectinase was determined by measuring the amount of reducing sugars released from a pectin solution using the 3,5-dinitrosalicylic acid (DNS) method proposed by Miller [51]. The DNS reagent is composed of 2-hydroxy-3,5-dinitrobenzoic acid (1% *w*/*v*), potassium sodium tartrate (30% *w*/*v*), and sodium hydroxide (1% *w*/*v*). The substrate solution was prepared with citric pectin (0.25% *w*/*v*) in citrate buffer (50 mM, pH 3.8).

In a typical assay, 0.1 mL of free PeL solution or 0.020 g of wet immobilized enzyme (PeL@PA6) was mixed with 0.90 mL of substrate solution and incubated at 50 °C for 15 min. Subsequently, 1 mL of DNS solution was added to the reaction mixture; the vials were kept in a water bath at 90 °C for 10 min and then cooled in an ice bath for 15 min to stop the reaction. Then, the dark yellow solution was measured at 540 nm using conventional UV-visible spectroscopy (Shimadzu UV-1900i). The blank consisted of 1 mL of DNS and 1 mL of buffer solution. The amount of reducing sugar released was quantified using the standard calibration curve for D-galacturonic acid. Enzymatic activity in units (U) was expressed as μmol of D-galacturonic acid equivalents released per minute under the assay conditions, according to Equation (2):

(2) $$A(U)=\frac{C}{T}$$

were C is the concentration of reducing sugars (μmol/mL) obtained from the calibration curve, and T is the reaction time (min).

##### Calibration Curve

The calibration curve was prepared using D-galacturonic acid as the standard. Standard solutions in the range of 0.28–2.83 μmol/mL were prepared in citrate buffer (50 mM, pH 3.8). A total of 1 mL of each standard was heated to 50 °C and mixed with 1 mL DNS reagent under the same conditions as described in Section Activity Assay.

##### Determination of Immobilization Efficiency and the Activity Recovery

The activity of the supernatants was also determined using the DNS method. These results were used to calculate the immobilization efficiency and the activity recovery. The immobilization efficiency (IE, Equation (3)) represents the fraction of the expected activity that was effectively retained in the immobilized form. The activity recovery (AR, Equation (4)) indicates the proportion of the initial total activity preserved after immobilization. They were calculated as defined by Sheldon and van Pelt [52] and further detailed by Boudrant et al. [53].

IE was calculated as the ratio of observed immobilized activity to the theoretical activity of the bound protein, assuming unchanged specific activity, i.e.,

(3) $$IE\left(\%\right)=\frac{{SA}_{imm}}{{SA}_{theor}}\times 100=\frac{AR}{IY}\times 100$$

wherein ${SA}_{imm}$ is the observed specific activity of the immobilized enzyme (U/mg), and ${SA}_{theor}$ is the theoretical activity of the bound enzyme (U/mg) determined as the product of the amount of bound protein and the initial specific activity of the free enzyme. 

AR was determined as the ratio of observed immobilized activity to the initial total enzyme activity, i.e.,

(4) $$AR\left(\%\right)=\frac{A_{imm}}{A_{0}}\times 100$$

wherein $A_{0}$ is the total enzymatic activity of the starting enzyme solution (U) and $A_{imm}$ is the activity measured for the immobilized complex (U). 

#### 3.2.5. Influence of Ethanol on Enzymatic Activity

The influence of ethanol on the activity of the free PeL or its immobilized complexes was determined by the DNS method, under the same conditions described above in Section Activity Assay. For this purpose, different percentages of ethanol (5, 10, and 15%, *v*/*v*) were added to both the substrate and the blank samples. The solutions obtained after the reaction with DNS were analyzed by UV/VIS spectroscopy.

#### 3.2.6. Influence of Glucose on Enzymatic Activity

The activity of free and immobilized lyophilized PeL was determined in the presence of different percentages of glucose (5, 10, and 15%, *w*/*v*). The glucose was added to the substrate solution, then 0.90 mL of this solution was added to 0.10 mL of PeL, or 0.020 g of PeL@PA6 was heated to 50 °C and mixed with the DNS reagent under the same conditions as the activity assay described in Section Activity Assay. The blank samples consisted of a solution of DNS, buffer solution, and different percentages of glucose. 

#### 3.2.7. Kinetic Studies

The kinetic parameters $V_{max}$ and $K_{m}$ for all PeL and PeL@PA6 samples were determined by applying the activity assay for different pectin concentrations ranging from 0.1 to 7 mg/mL. Based on this data, the Michaelis–Menten hyperbolic curves (Equation (5)) and the Lineweaver–Burk linear plots (Equation (6)) were constructed. The kinetic parameters were calculated from both equations by non-linear and linear regression calculations using OriginPro software (version 9.8.0.200, OriginLab Corporation, Northampton, MA, USA).

(5) $$V_{0}=\frac{V_{max}.[S]}{K_{m}+[S]}$$

(6) $$\frac{1}{V_{0}}=\frac{K_{m}}{V_{max}}\times \frac{1}{\left[S\right]}+\frac{1}{V_{max}}$$

#### 3.2.8. Clarification Studies

Clarification of white wine and rosé musts was performed at room temperature using free PeL and PeL@PA complexes obtained at various pH concentrations. In total, 14 mL of rosé or white must, containing 2 mg/mL pectin, was placed in Falcon tubes, where a certain amount of free or immobilized enzyme was added—180 µL of PeL solution containing 0.1 mg of free enzyme (pH 5–8) or the corresponding amount of immobilized enzyme containing the same amount of enzyme. Clarification was determined by measuring turbidity values expressed in nephelometric turbidity units (NTU) and recorded every 20 min for a total period of 3–9 h.

#### 3.2.9. Pectin Test

After clarification, pectin tests were performed to verify whether the clarification process was complete. For this test, 5 mL of acidified alcohol (5% *v*/*v* HCl in ethanol) was added to 2.5 mL of clarified must. The mixture was gently shaken, allowed to stand for 10 min, and then visually inspected. The formation of insoluble flakes indicated the presence of residual pectin, while a clear liquid confirmed complete clarification.

#### 3.2.10. Color Analyses of Must Samples 

The color of the must samples was analyzed by UV/VIS spectroscopy, according to the method OIV-MA-AS2-780 07B [54]. These analyses were made to evaluate any color changes resulting from enzymatic clarification. The color of the must was measured at three specific wavelengths: 420 nm (absorption of yellow), 520 nm (absorption of red), and 620 nm (absorption of blue). The baseline used was double-distilled water.

The color intensity I that reflects the overall amount of coloring compounds was calculated using Equation (7). The tonality T (also referred to as hue), was established by Equation (8). The percentages of yellow (Y, Equation (9)), red (R, Equation (10)), and blue (B, Equation (11)) color components were also calculated.

(7) $$I(a.u.)=A_{420}+A_{520}+A_{620}$$

(8) T(a.u.)=A420A520

(9) $$Y(\%)=\frac{A_{420}}{I}\times 100$$

(10) $$R(\%)=\frac{A_{520}}{I}\times 100$$

(11) $$B(\%)=\frac{A_{620}}{I}\times 100$$

where $A_{420}$, $A_{520}$, and $A_{620}$ represent the absorbance values at 420 nm, 520 nm, and 620 nm, respectively.

The total polyphenolic index (TPI), hydroxycinnamic acids (HCA) and flavanols (FLAV) were measured using the absorbances at 280, 320 and 365 nm, respectively, after sample dilution 1:10 with distilled water for white must and 1:5 with distilled water for rose must. More details about these measurements can be found elsewhere [43].

#### 3.2.11. Reusability Studies by Clarification

The PeL@PA6 complexes, each containing 0.1 mg of enzyme, were used for clarification of rosé/white must (15 mL) in two consecutive cycles. Turbidity was monitored every hour for the must until ≈20 NTU. After each cycle, the PeL@PA6 complexes were recovered via centrifugation and washed twice with 1 mL of deionized water. Their clarification capacity was tested again under the same conditions with a fresh must sample.

#### 3.2.12. Reusability Studies by Activity Tests

For this study, PeL@PA6 complexes (0.020 g) were incubated with 0.90 mL of citric pectin substrate at 50 °C for 15 min. After incubation, the liquid phase was separated from the immobilized complexes to terminate the reaction. Subsequently, 1 mL of DNS reagent was added to the supernatant to chemically inactivate any potential leached enzyme and stabilize the samples for storage in the dark until further processing. The complexes were then washed twice with 1 mL of deionized water to remove residual substrate before starting a new cycle. This procedure was repeated for five cycles. After the final cycle, all collected vials containing DNS were heated at 90 °C for 10 min to develop the color, cooled in ice water, and analyzed by UV/VIS spectroscopy. Residual activity was determined by comparing subsequent cycles to the first cycle, the latter being considered 100%.

#### 3.2.13. Storage Stability Studies

The PeL and PeL@PA6 were stored at 4 °C in the same conditions used during immobilization (pH 5 through 8) for up to four months. Samples were taken after 1, 2, 3, and 4 weeks, and again after 2, 3, and 4 months, and their residual activity was determined using the DNS method, as described above in Section Activity Assay. The activity measured immediately after immobilization was taken as 100% and used as the reference value.

## 4. Conclusions

Lyophilized pectinase PeL was successfully immobilized on PA6 MPs through a simple adsorption-based procedure, varying the pH of the immobilization medium. An enzyme concentration of 0.5 mg mL^−1^ was identified as optimal for preparing stable and catalytically active conjugates. Thermal characterization by TGA demonstrated that the immobilized PeL modified the degradation behavior of the PeL@PA6 complexes, producing a more gradual thermal decomposition as compared to the neat PA6 MPs.

UV-CD analysis revealed that the secondary structure of pectinase is strongly influenced by pH, with the enzyme displaying the greatest conformational stability in the pH range 6–7. In most of the cases, the immobilized enzyme retained significant catalytic competence, as reflected by higher specific activities and immobilization efficiencies, as well as the occurrence of hyperactivation effects under certain conditions.

The catalytic performance of both free and immobilized enzymes was affected by components relevant to enological environments. Glucose acted as an inhibitor of pectinase activity, whereas ethanol promoted moderate activation within the tested concentration range, indicating that the enzyme exhibits good tolerance to ethanol-rich media. These results suggest that the immobilized enzyme is well suited for applications in wine-processing environments.

Kinetic analysis showed that immobilization generally resulted in higher apparent $K_{m}$ values while $V_{max}$ remained comparable to, or even higher than that of the free enzyme in some cases. These changes are consistent with diffusional limitations and restricted substrate accessibility associated with the porous structure of the PA6 support rather than intrinsic alterations of the catalytic mechanism.

Application tests using industrial white and rosé wine musts demonstrated the practical relevance of the immobilized system. Although clarification required slightly longer times than with the free enzyme, the immobilized PeL preserved the color and phenolic integrity of the musts, maintaining product quality. Importantly, the immobilized biocatalysts showed good operational stability, displaying retained activity of 40% in up to five consecutive cycles. The reusability of the complexes in real wine musts proved industrially viable in two consecutive cycles. After four months of storage, the immobilized complexes displayed higher remaining activities than the respective free enzymes.

Although the immobilized PeL@PA6 biocatalysts demonstrated promising catalytic performance and operational stability, the mechanisms responsible for the gradual loss of activity upon repeated use, including the possible contribution of enzyme desorption and irreversible deactivation, were not investigated in detail. Furthermore, the comparison between the free and immobilized enzyme was focused primarily on catalytic performance and kinetic behavior under the investigated conditions. Future studies should include a more comprehensive comparative analysis of the structural stability, deactivation mechanisms, and long-term operational performance of both enzyme forms, as well as optimization of the immobilization procedure to further improve catalyst durability and reusability.

Overall, the results demonstrate that PA6 MPs constitute robust and effective supports for pectinase immobilization. The combination of structural stability, catalytic performance, and operational durability highlights the potential of this immobilization strategy for practical applications in wine clarification and other biotechnological processes related to pectin elimination. 

## Figures and Tables

**Table 1:** Secondary structure estimation of lyophilized pectinase (PeL) before and after immobilization (SN_PeL) at different pH values. The data presented correspond to the average of all matching solutions for enhanced statistical representativeness.

Samples	α-Helix (%)	β-Sheet (%)	Turns (%)	Unordered (%)
PeL_pH5	−	−	−	−
SN_PeL_pH5	−	−	−	−
PeL_pH6	12.8	37.7	24.8	24.7
SN_PeL_pH6	25.8	11.4	28.2	34.4
PeL_pH7	15.3	33.9	17.1	33.7
SN_PeL_pH7	20.7	20.6	23.4	35.3
PeL_pH8	4.0	33.8	26.5	35.8
SN_PeL_pH8	23.1	12.4	31.8	32.8

**Table 2:** TGA of polyamide microparticles.

Sample	$T_{in},{}^{\circ}C$ ^a^	$T_{max,}{}^{\circ}C$ ^b^			$V_{max}^{d},\%/{}^{\circ}C$ ^c^			Residue at 600 °C, %
		I	II	III	I	II	III	
PA6	266.95	335.38	-	-	2.76	-	-	1.546
PeL@PA6_pH5	311.36	330.96	-	-	2.44	-	-	9.161
PeL@PA6_pH6	396.69	450.28	-	-	1.133	-	-	10.53
PeL@PA6_pH7	333.65	346.72	387.57	436.21	0.4466	0.7211	0.7536	6.708
PeL@PA6_pH8	305.41	340.60	423.47	-	1.12	0.296	-	11.77

^a^ $T_{in}=$ Temperature of initial degradation obtained from integral TGA curves; ^b^ $T_{max}=$ Maximum degradation temperature obtained from the peak of the 1st derivative of the TGA curves; ^c^ $V_{max}^{d}=$ Maximum degradation rate at $T_{max}$ obtained from the first derivative of the TGA curves.

**Table 3:** Activity parameters of free and immobilized lyophilized pectinase as a function of pH.

Sample Designation	Protein Content (mg)	Immobilization Yield (IY * (%))	Specific Activity (U/mg Protein)	Immobilization Efficiency (IE(%))	Activity Recovery (AR(%))
PeL_pH5	2.500	−	2.146	−	−
PeL@PA6_pH5	0.296	11.8	4.646	233.8	27.6
PeL_pH6	2.500	−	2.097	−	−
PeL@PA6_pH6	0.596	23.8	2.400	114.6	27.3
PeL_pH7	2.500	−	2.816	−	−
PeL@PA6_pH7	0.825	33.0	2.213	78.6	25.9
PeL_pH8	2.500	−	2.434	−	−
PeL@PA6_pH8	0.489	19.6	2.719	111.7	21.9

* See also Figure 2c.

**Table 4:** Kinetic parameters of free and immobilized pectinase.

Sample	Parameters *	Non-Linear MM Fitting	LB Double Linearization
PeL_pH5	$V_{max}\;(mM\;min^{-1}{mg}^{-1})$	35.327 ± 1.399	33.478 ± 3.746
	$K_{m}\;(mg\;mL^{-1})$	0.654 ± 0.086	0.398 ± 0.044
	$Adj.R^{2}$	0.988	0.998
PeL@PA6_pH5	$V_{max}\;(mM\;min^{-1}{mg}^{-1})$	93.061 ± 9.136	73.368 ± 6.243
	$K_{m}\;(mg\;mL^{-1})$	2.814 ± 0.484	2.006 ± 0.179
	$Adj.R^{2}$	0.992	0.998
PeL_pH6	$V_{max}\;(mM\;min^{-1}{mg}^{-1})$	40.159 ± 2.870	47.801 ± 5.670
	$K_{m}\;(mg\;mL^{-1})$	0.563 ± 0.143	0.906 ± 0.110
	$Adj.R^{2}$	0.955	0.999
PeL@PA6_pH6	$V_{max}\;(mM\;min^{-1}{mg}^{-1})$	37.456 ± 2.712	27.420 ± 2.482
	$K_{m}\;(mg\;mL^{-1})$	1.916 ± 0.283	1.119 ± 0.111
	$Adj.R^{2}$	0.991	0.992
PeL_pH7	$V_{max}\;(mM\;min^{-1}{mg}^{-1})$	49.707 ± 4.462	52.301 ± 8.483
	$K_{m}\;(mg\;mL^{-1})$	0.789 ± 0.215	1.015 ± 0.169
	$Adj.R^{2}$	0.955	0.998
PeL@PA6_pH7	$V_{max}\;(mM\;min^{-1}{mg}^{-1})$	36.806 ± 4.386	21.386 ± 1.815
	$K_{m}\;(mg\;mL^{-1})$	3.325 ± 0.655	1.416 ± 0.129
	$Adj.R^{2}$	0.990	0.996
PeL_pH8	$V_{max}\;(mM\;min^{-1}{mg}^{-1})$	52.068 ± 2.969	50.050 ± 7.184
	$K_{m}\;(mg\;mL^{-1})$	0.950 ± 0.151	0.988 ± 0.144
	$Adj.R^{2}$	0.985	0.998
PeL@PA6_pH8	$V_{max}\;(mM\;min^{-1}{mg}^{-1})$	69.480 ± 7.691	48.123 ± 6.647
	$K_{m}\;(mg\;mL^{-1})$	3.150 ± 0.587	1.836 ± 0.260
	$Adj.R^{2}$	0.991	0.994

Note: * $V_{max}$, normalized by the weight of the protein, in fact, represents the catalytic turnover $K_{cat}$. It denotes the number of substrate molecules converted into product per milligram of enzyme per minute, when the enzyme is saturated with substrate. MM = Michaelis–Menten; LB = Lineweaver-Burk.

## Data Availability

All the data generated during this research are presented in the manuscript and in the Supplementary Materials.

## Supplementary Materials

The following supporting information can be downloaded at https://www.mdpi.com/article/10.3390/molecules31162930/s1. Figure S1: CD UV curves and their fits of at pH 6; Figure S2: CD UV curves and their fits of at pH 7; Figure S3: CD UV curves and their fits of at pH 8; Figure S4: Pectin test with must samples before and after clarification; Table S1: pH of must before and after the end of clarification time; Table S2: Estimated white wine must color and composition measures after clarification with free and immobilized PeL; Table S3: Estimated rosé must color and composition measures after clarification with free and immobilized PeL; Figure S5: Chemical reactions occurring during AAROP of ε-caprolactam (ECL) to neat PA6 MP.

## Abbreviations

The following less common abbreviations are frequently used in this manuscript:

AAROP	Activated anionic ring-opening polymerization
AR	Activity recovery
CD	Circular dichroism
DNS	3,5-dinitrosalicylic acid
IE	Immobilization yield
MP	Microparticles
NTU	Nephelometric turbidity unit
PeL	Lyophilized pectinase
PeL@PA6	PeL immobilized on PA6
pI	Isoelectric point
SN	Supernatant
