# Supplementary material for: Pectinase Immobilization on Porous Polyamide Microparticles: Characterization, Operational Stability and Application in Wine Clarification
Source: Molecules. 2026 Aug 21;31(16):2930. doi: 10.3390/molecules31162930 (PMC13515607; doi:10.3390/molecules31162930)
Supplement: Supplementary file 1 [file molecules-31-02930-s001.zip › molecules-4436329-supplementary.pdf]

molecules

MDPI

Article

Pectinase Immobilization on Porous Polyamide Microparticles: Characterization, Operational Stability and Application in Wine Clarification

Sandra C. Oliveira, Nadya V. Dencheva and Zlatan Z. Denchev *

IPC—Institute for Polymers and Composites, Campus of Azurém, University of Minho, 4800-058 Guimarães, Portugal; b8223@dep.uminho.pt (S.C.O.); nadiad@dep.uminho.pt (N.V.D.)

* Correspondence: denchev@dep.uminho.pt

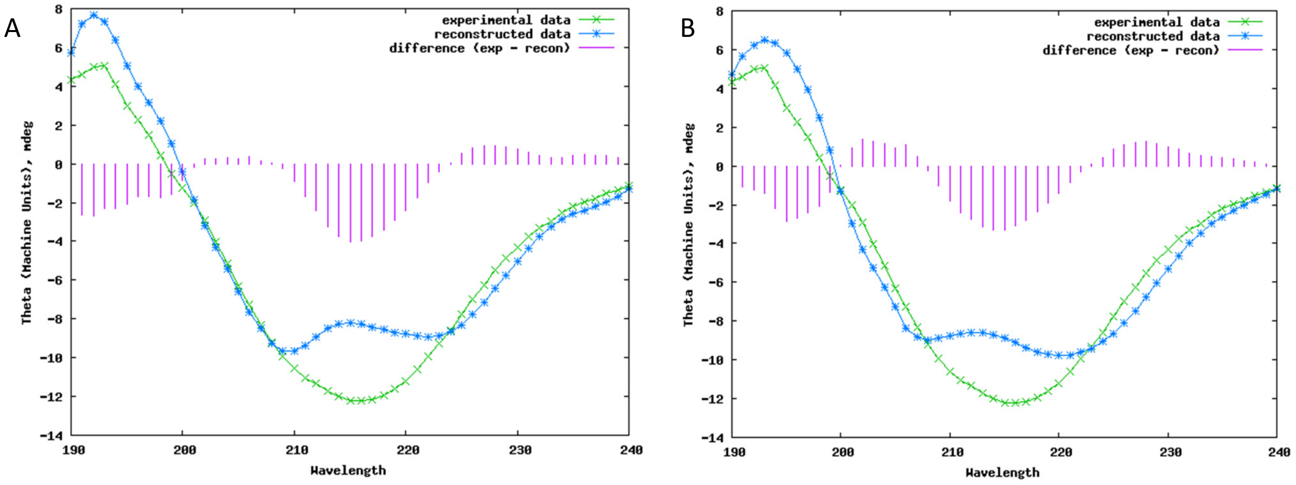

Figure S1. CD UV curves and their fits of at pH 6: A) free PeL, B) its supernatants after immobilization

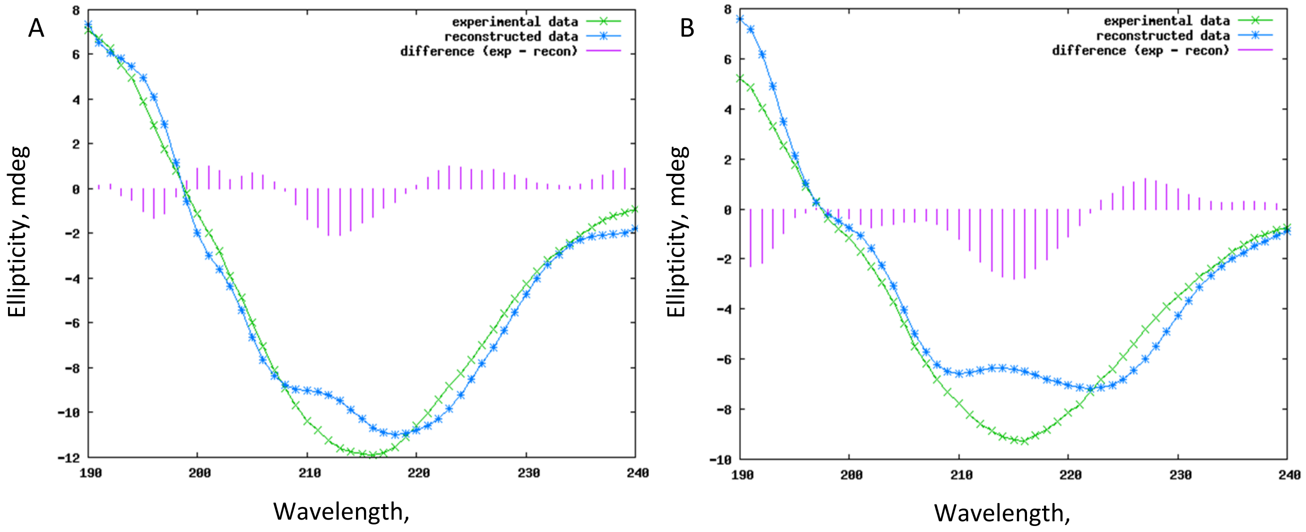

Figure S2. CD UV curves and their fits of at pH 7: A) free PeL, B) its supernatants after immobilization

molecules

MDPI

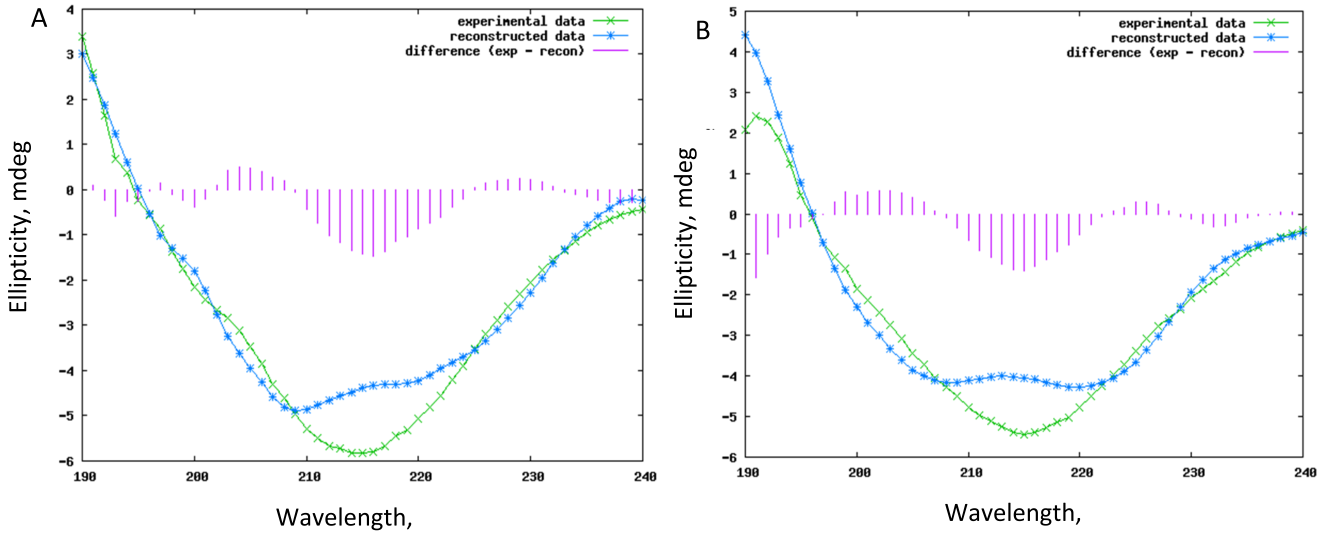

Figure S3. CD UV curves and their fits of at pH 8: A) free PeL, B) its supernatant after immobilization

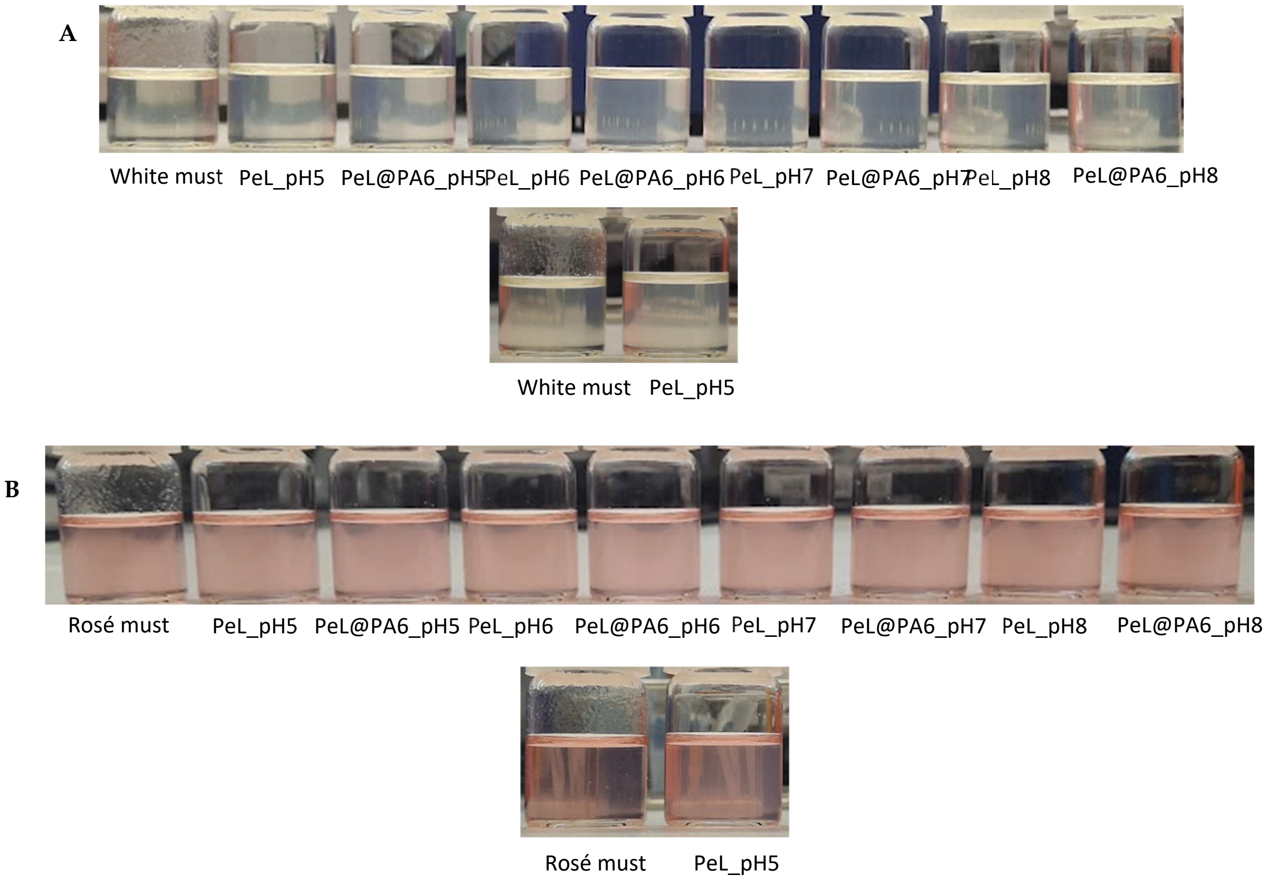

Figure S4. Pectin test with must samples before and after clarification: A – white wine must: B – rosé wine must. Visible aggregates (gelatinous floccules) were observed exclusively in the untreated must samples (controls).

molecules

MDPI

Table S1. pH of must before and after the end of clarification time.

Must	White must	Rosé must
Untreated Must	3.19	3.44
PeL_pH5	3.14	3.41
PeL@PA6_pH5	3.13	3.39
PeL_pH6	3.12	3.38
PeL@PA6_pH6	3.13	3.40
PeL_pH7	3.10	3.39
PeL@PA6_pH7	3.11	3.44
PeL_pH8	3.14	3.41
PeL@PA6_pH8	3.12	3.41

Table S2. Estimated white wine must color and composition measures after clarification with free and immobilized PeL. Two tests were performed for each sample. For addreviation designation see the table at the end of the article.

White must	***BI*** **(*a.u.*)**	***I*** **(*a.u.*)**	***T*** **(*a.u.*)**	***Y*** (%)	***R*** (%)	***B*** (%)	***TPI*** **(*a.u.*)**	***HCA*** **(*a.u.*)**	***FLAV*** **(*a.u.*)**
Untreated Must	0.2825	0.417	2.94	67.75	23.02	9.23	8.88	4.305	1.005
PeL_pH5	0.137	0.176	4.57	77.84	17.05	5.11	8.04	3.86	0.73
PeL@PA6_pH5	0.128	0.165	4.57	77.58	16.97	5.45	8.03	3.89	0.745
PeL_pH6	0.137	0.1765	4.49	77.62	17.28	5.10	7.85	3.77	0.715
PeL@PA6_pH6	0.129	0.168	4.30	76.79	17.86	5.36	7.215	3.51	0.685
PeL_pH8	0.144	0.188	4.24	76.60	18.09	5.32	7.66	3.69	0.72
PeL@PA6_pH8	0.14	0.183	4.24	76.50	18.03	5.46	7.65	3.705	0.72
PeL_pH7	0.14	0.181	4.38	77.35	17.68	4.97	9.11	4.395	0.85
PeL@PA6_pH7	0.135	0.175	4.35	77.14	17.71	5.14	8.305	4.01	0.76

Table *S3*. Estimated rosé must color and composition measures after clarification with free and immobilized PeL. Two tests were performed for each sample. For addreviation designation see the table at the end of the article.

Rosé must	***BI*** **(*a.u.*)**	***I*** **(*a.u.*)**	***T*** **(*a.u.*)**	***Y*** (%)	***R*** (%)	***B*** (%)	***TPI*** **(*a.u.*)**	***HCA*** **(*a.u.*)**	***FLAV*** **(*a.u.*)**
Untreated Must	0.5705	0.93	1.99	61.29	30.75	7.96	15.24	7.835	1.65
PeL_pH5	0.42	0.679	1.90	61.86	32.55	5.60	16.05	8.135	1.495
PeL@PA6_pH5	0.39	0.626	1.94	62.30	32.11	5.59	15.16	7.84	1.425
PeL_pH6	0.425	0.692	1.86	61.42	32.80	5.78	15.475	7.865	1.45
PeL@PA6_pH6	0.378	0.606	1.94	62.38	32.18	5.45	14.975	7.665	1.41
PeL_pH8	0.422	0.683	1.90	61.79	32.50	5.71	15.48	7.895	1.45
PeL@PA6_pH8	0.436	0.701	1.93	61.34	32.24	6.42	15.025	7.665	1.44
PeL_pH7	0.421	0.685	1.87	61.46	32.85	5.69	15.96	8.08	1.465
PeL@PA6_pH7	0.436	0.708	1.90	61.58	32.49	5.93	15.33	7.885	1.45

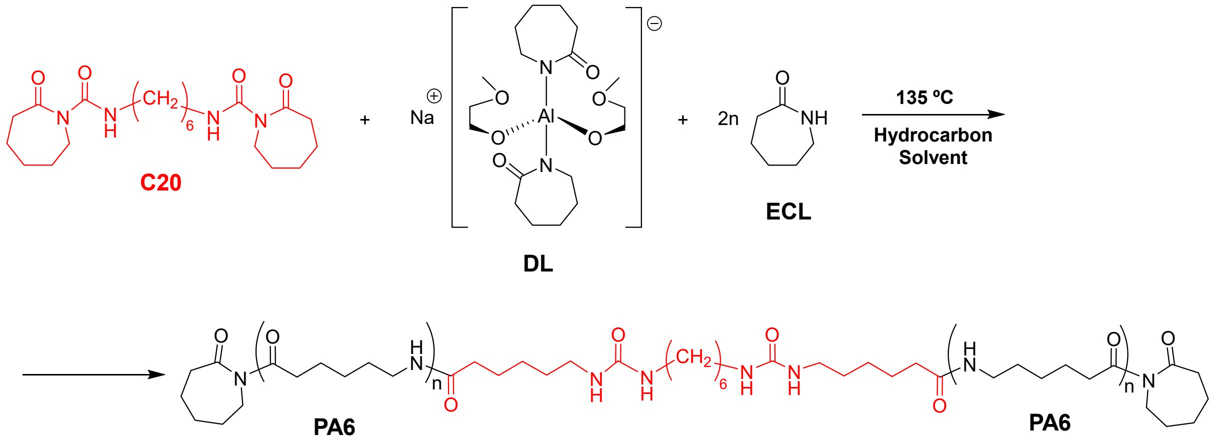

Figure S5. Chemical reactions occurring during AAROP of ε-caprolactam (ECL) to neat PA6 MP. The active substance of the AAROP activator is designated as C20; The chemical structure of the AAROP initiator dicaprolactamato-bis-(2-methoxyethoxo)-aluminate (DL) is presented wherein R = $OCH_2CH_2OCH_3$.
